# Critical band-to-band-tunnelling based optoelectronic memory

**DOI:** 10.1038/s41377-025-01756-7

**Published:** 2025-02-07

**Authors:** Hangyu Xu, Runzhang Xie, Jinshui Miao, Zhenhan Zhang, Haonan Ge, Xuming Shi, Min Luo, Jinjin Wang, Tangxin Li, Xiao Fu, Johnny C. Ho, Peng Zhou, Fang Wang, Weida Hu

**Affiliations:** 1 State Key Laboratory of Infrared Physics, Shanghai Institute of Technical Physics, Chinese Academy of Sciences, 500 Yu Tian Road, 200083, Shanghai, China; 2 University of Chinese Academy of Sciences, Beijing, China; 3 ASIC & System State Key Laboratory, School of Microelectronics, Fudan University, Shanghai, China; 4 Shanghai Research Institute for Intelligent Autonomous Systems, Tongji University, 200092, Shanghai, China; 5 Department of Materials Science and Engineering and State Key Laboratory of Terahertz and Millimeter waves, City University of Hong Kong, Kowloon Tong, Hong Kong SAR, China

**Keywords:** Imaging and sensing, Photonic devices

## Abstract

Neuromorphic vision hardware, embedded with multiple functions, has recently emerged as a potent platform for machine vision. To realize memory in sensor functions, reconfigurable and non-volatile manipulation of photocarriers is highly desirable. However, previous technologies bear mechanism challenges, such as the ambiguous optoelectronic memory mechanism and high potential barrier, resulting in a limited response speed and a high operating voltage. Here, for the first time, we propose a *critical band-to-band tunnelling (BTBT)* based device that combines sensing, integration and memory functions. The nearly infinitesimal barrier facilitates the tunnelling process, resulting in a broadband application range (940 nm). Furthermore, the observation of dual negative differential resistance (NDR) points confirms that the *critical BTBT* of photocarriers contributes to the sub-microsecond photomemory speed. Since the photomemory speed, with no motion blur, is important for motion detection, the critical BTBT memory is expected to enable moving target tracking and recognition, underscoring its superiority in intelligent perception.

## Abstract (web summary)

Critical band-to-band tunneling is demonstrated at the van der Waals interface, with potential applications in optoelectronic memory.

## Graphical abstract

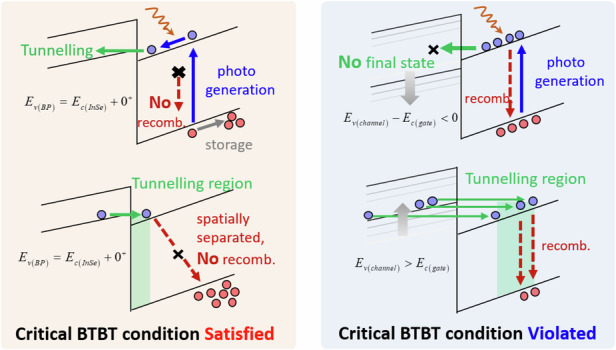

## Introduction

The advent of machine vision has fundamentally changed human life^1–4^, and proven technologies are merging and leading the transition to Industry 4.0^5^. During the transition, extensive unprocessed images are emerging, posing significant challenges to the image processing systems^6–22^. In typical designs of processing systems, the sensory units are physically separated from memory and computing units. Visual information is first sent to binary memory via high-power analogue-to-digital conversion (ADC) and then processed by computing units. A large amount of data shuffling between units renders severe speed incongruity and the power-consumption dilemma^23–26^. To efficiently address the proliferation of visual information, approaches must be developed to integrate module functions that can reduce the redundant data shuffle from sensing to computing units.

To date, much work has been devoted to integrating multiple functions in one device and developing a data-centric approach. Neuromorphic photonics for vision sensing is now widely developing, including optoelectronic synapse^27^, metasurface^15,19,26^, integrated photonics^21,28^, electric memory array^20^, etc. Among them, a promising solution, optoelectronic memory, has been proposed, which can be programmed by optical stimuli and read out by electrical operations^29–32^. Technologies based on photogating and the Fowler–Nordheim tunnelling mechanism may be suitable. However, the ambiguous trap energy level locations and high barrier lead to a limited response speed and a high operating voltage^33–36^.

Here, band-to-band-tunnelling (BTBT)-based optoelectronic memory is realized based on black phosphorus (BP) and indium selenium (InSe). With deliberate band alignment, our device satisfies the *critical BTBT condition*, exhibiting cumulative photomemory current and a low operating voltage. Due to the infinitesimal barrier, the operating wavelength of the critical BTBT memory, i.e., BP on the InSe device, is extended to the near-infra-red region (940 nm). In addition, the calculated dual negative differential resistance (NDR) points are consistent with the experimental results, demonstrating the emergence of the BTBT process. The corresponding hole memory and electron tunnelling processes are elaborated through time evolution simulation results. Finally, noticing that a poor memory speed leads to severe motion blur, we evaluate its impact on motion detection based on a comparison. With motion blur eliminated, the critical BTBT memory significantly improves both moving target tracking and recognition. The critical BTBT memory, embedded with sensing, integration and memory functions, proposes a better solution for achieving a sub-microsecond photomemory speed, showcasing superiority in intelligent perception (Fig. S1).

## Results

### Device structure and critical BTBT mechanisms

**Fig. 1:**
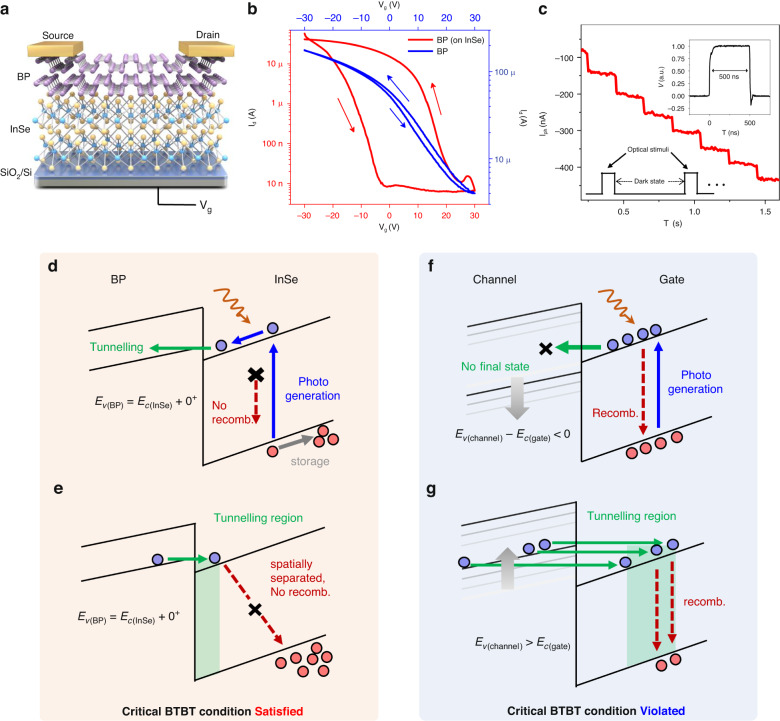
***Critical BTBT*** **in BP on InSe devices. a** Schematic diagram of the critical BTBT memory. **b** Transfer characteristics of the critical BTBT memory (red line) and a BP transistor (blue line). **c** Negative photomemory characteristics of the critical BTBT memory in response to multiple 520 nm laser pulses with a duration of 500 ns. Inset: Demonstration of the 500 ns laser pulse width. In **d–g**, band alignments are depicted at *V*_g_ < 0 with a constant *V*_d_. **d** Photoresponse mechanism under the *critical BTBT condition*. Photo-injected electron-hole pairs are separated by a tilted energy band in InSe, and electrons further tunnel into BP, preventing recombination. **e** Mechanism of hole recombination suppression under the *critical BTBT condition*. The region where electrons in BP can tunnel to InSe is separated in space from the region where holes are stored, avoiding the recombination with stored holes in InSe. **f** Schematic of the photoresponse mechanism when the valence band in the channel is below the *critical BTBT condition*. Electrons in the conduction band in the gate have no final state to tunnel to. Both electrons and holes are stored in the gate, resulting in severe recombination. **g** Recombination mechanism of stored holes when the valence band in the channel is above the *critical BTBT condition*. A higher valence band in the channel allows the tunnelling of electrons to deep into the gate. The tunnelling region overlaps with the region occupied by stored holes, causing recombination. **f** and **g** refer to the energy band diagrams of other heterogeneous interfaces that do not satisfy *critical BTBT conditions*

Figure 1a shows a schematic of the three-terminal critical BTBT memory with source and drain contacts on the BP channel, while the InSe layer is floating and covered by the BP channel as a gate. p^++^ Si serves as the back gate (*V*_g_). InSe is chosen as the gate because the current of InSe-based heterojunctions is limited by the interface carrier blocking effect^29,30,37–40^, increasing the RC time constant (Supplementary Sections 3 and 4). With an enlarged RC time constant, the memory window is remarkably extended, as shown in Fig. 1b. The memory window of the critical BTBT memory reaches 25 V in a counterclockwise direction; in contrast, the memory window of individual BP transistors is relatively narrow. In addition, when the channel material BP is replaced with other materials, such as WSe_2_ and MoTe_2_, with similar bipolar characteristics to BP, the memory window is similarly enlarged (Supplementary Section 5), demonstrating the potential for memory applications.

However, there is a substantial difference in the photoresponse behaviours of these devices. With a negative *V*_g_ and the application of sub-microsecond level optical stimuli in fixed intervals, only the critical BTBT memory (BP on InSe device) anomalously generates a cumulative and negative photocurrent and preserves it even in the dark state, as shown in Fig. 1c, which is defined as the photomemory current (the performances under different *V*_g_ values and different wavelengths are shown in Supplementary Section 6). Conversely, the photoresponses of the MoTe_2_ on InSe and WSe_2_ on InSe devices are all positive and stable with no memory characteristics. After the exclusion of the interface defect artefact (Fig. S14), based on the band alignment calculation, the *critical BTBT* mechanism is proposed to interpret the unique optoelectronic memory characteristics of the critical BTBT memory. *Critical BTBT* satisfies the two prerequisites for realizing InSe-based optoelectronic memory: (1) fast photocarrier separation and (2) recombination suppression. To reduce the tunnelling of equilibrium carriers, the types of quasiparticles in the bands of the initial and final states should be different. Take the case of hole storage as an example. First, accelerating the photocarrier separation through established channels is crucial in the light state. Note that the interface-blocking effect significantly suppressed the diffusion and drift currents across the interface. A promising channel to cross the interface is BTBT, which enables photogenerated carriers to separate rapidly into adjacent materials under an electric field. Considering the final states to be tunnelled to (Fig. 1d, f), the valence band maximum of the channel ${E}_{{\rm{v}}({\rm{c}}{\rm{h}}{\rm{a}}{\rm{n}}{\rm{n}}{\rm{e}}{\rm{l}})}$ should be above the conduction band minimum of the gate ${E}_{{\rm{c}}({\rm{g}}{\rm{a}}{\rm{t}}{\rm{e}})}$, i.e., ${E}_{{\rm{v}}({\rm{c}}{\rm{h}}{\rm{a}}{\rm{n}}{\rm{n}}{\rm{e}}{\rm{l}})} > {E}_{{\rm{c}}({\rm{g}}{\rm{a}}{\rm{t}}{\rm{e}})}$. Meanwhile, regarding the dark state, as tunnelling is a time-reversal symmetric process, the key is to suppress the undesired recombination caused by reverse BTBT. In an extremely thin gate (InSe layer), the spatial overlap between the electron tunnelling region and the hole storage region must be minimized. Given that the deepest tunnelling region for reverse tunnelling is mainly determined by ${E}_{{\rm{v}}({\rm{c}}{\rm{h}}{\rm{a}}{\rm{n}}{\rm{n}}{\rm{e}}{\rm{l}})}-{E}_{{\rm{c}}({\rm{g}}{\rm{a}}{\rm{t}}{\rm{e}})}$ (Fig. 1e, g and Supplementary Section 15), ${E}_{v({\rm{c}}{\rm{h}}{\rm{a}}{\rm{n}}{\rm{n}}{\rm{e}}{\rm{l}})}$ should be slightly higher than ${E}_{{\rm{c}}({\rm{g}}{\rm{a}}{\rm{t}}{\rm{e}})}$. Therefore, the optimal conditions to achieve both excellent photomemory efficiency and a long retention time should be

$$\Delta E={E}_{{\rm{v}}({\rm{c}}{\rm{h}}{\rm{a}}{\rm{n}}{\rm{n}}{\rm{e}}{\rm{l}})}-{E}_{{\rm{c}}({\rm{g}}{\rm{a}}{\rm{t}}{\rm{e}})}\to {0}^{+}$$

which we denote as the *critical BTBT condition*. From the Kelvin probe force microscopy (KPFM) characterizations in Fig. S2, the *critical BTBT condition* is consistent with the band alignment of the critical BTBT memory.

### Photomemory characteristics

**Fig. 2:**
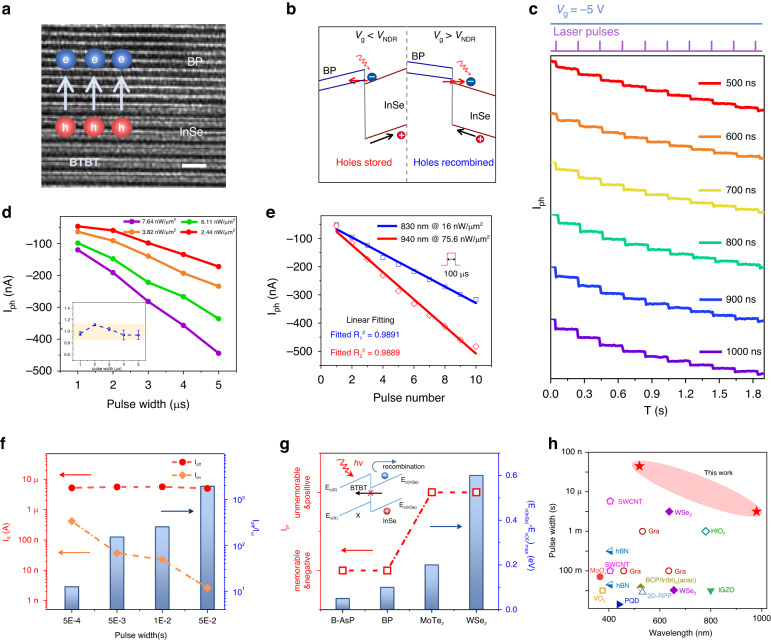
**Photomemory characteristics of critical BTBT memory. a** Cross-sectional TEM images of the overlapped region, showing a clean interface with an atomic sharpness, and the scale bar is 2 nm. **b** Corresponding band alignment of critical BTBT memory under different gate voltage. Red arrows represent the direction of BTBT, and black arrows represent the direction of photogenerated holes. **c** Negative photomemory current in response to 520 nm laser pulses, which are 500–1000 ns in duration with a 0.2 s interval. The photocurrent exhibits multiple states under cumulative laser pulse modulation. The critical BTBT memory works at *V*_d_ = 1 V and *V*_g_ = -5 V. **d** Modulation of the photomemory current by the laser pulse width under different intensities. Inset image: Statistical distribution of the light intensity dependence of the photomemory current for different pulse widths. **e** Linear variation in the negative photomemory current under cumulative NIR laser pulses with a 100 μs duration and a 1 s interval. The linear fit for the blue line (830 nm) is *y* = *a* × *x* + *b*, where *a* is −29, *b* is −38.5, and *R*_1_^2^ = 0.9891; the linear fit for the red line (940 nm) is *y* = *c* × *x* + *d*, where *c* is −47.9, *d* is −28.2, and *R*_2_^2^ = 0.9889. **f** Photomemory characteristics of the critical BTBT memory in response to 520 nm laser pulses. Red points indicate the initial state *I*_off_, and orange points represent the final state *I*_*on*_, which is read at 0.5 s after the laser pulse. The blue columns represent the ratio of *I*_off_ and *I*_on_. **g** Replace BP with other materials. The red square represents whether the photo response is positive or negative with memory characteristics. The blue column indicates the difference between *E*_C(InSe)_ and *E*_V*(X)*_, where *X* represents different materials. Inset image: For the device without the BTBT pathway, the photogenerated electrons will be recombined after light illumination, showing a positive response. **h** Comparison of the memorable pulse width and corresponding wavelength among reported optoelectronic memories. The references for the selected work can be found in Supplementary Table 1

High-resolution scanning transmission electron microscopy (STEM) confirms a clear and sharp interface at the BP/InSe junction in Fig. 2a. With the *critical BTBT condition* satisfied, critical BTBT memory exhibits negative photomemory characteristics when *V*_g_ is below *V*_NDR_, which is the gate voltage when negative differential resistance (NDR) occurs. The holes are stored in the valence band potential wall, which is accompanied by negative gate voltage, rendering a non-volatile photomemory. While the memory characteristics are constrained when *V*_g_ is above *V*_NDR_, the BTBT pathway is closed with no final states to tunnel to (Supplementary Section 15). Thus, as long as the gate voltage is appropriate, the BTBT channel opens, and the infinitesimal barrier facilitates photogenerated electron tunnelling. Figure 2c shows that when a fixed *V*_g_ = −5 V is applied, accompanied by laser stimuli, the negative photomemory current progressively varies with the multiple applied laser stimuli. The device shows a sub-microlevel second negative photomemory current (Supplementary Sections 9, 10). Figure 2d shows the high linearity in multiple dimensions by fitting the formula $I={P}^{\alpha }$. The extracted values for *α* are close to 1, demonstrating that the photomemory current can be linearly varied by both the power intensity and pulse width. Furthermore, as shown in Fig. 2e and f, the device shows a broadband response to near-infra-red rays (NIR) and a high dynamic range. The endurance and time stability are shown in Supplementary Sections 11 and 12.

In contrast, when an arbitrary prerequisite of the *critical BTBT condition* is violated, the photomemory current disappears. We conducted a series of comparative experiments with the same device structures but different materials, the corresponding detailed discussions are provided in Supplementary Section 7. Only the band alignment of BP/InSe exhibits broken gap configuration; the others demonstrate staggered gap configuration. First, for the devices with different channel materials (InSe still working as the gate), the drift and diffusion currents across the interface are significantly suppressed, and the first prerequisite of fast carrier separation is violated due to the lack of a BTBT channel. Unseparated electron-hole pairs are recombined in InSe under dark states, and the positive photoresponse is only attributed to the channel materials, as shown in Fig. 2g. Second, for the devices with different gate materials (BP still working as the channel), the drift and diffusion currents are, in contrast, unignorable^41^, contrary to the second prerequisite of recombination suppression. Thus, although driven by the built-in electric field of the PN junction, photogenerated holes remain in the gate, leading to a negative photoresponse in the light states; the inevitably elevated dark current prohibits the photoresponse from being memorized in the dark states. In addition, as b-AsP/InSe demonstrates similar band alignment with BP/InSe, it also exhibits negative photomemory current, as shown in Fig. 2g and Supplementary Section 5. The figures-of-merits of various optoelectronic memory devices are compared in Fig. 2e, showing that the critical BTBT memory has an outstanding photomemory speed and a broadband response.

### Experimental observation of dual NDR points

**Fig. 3:**
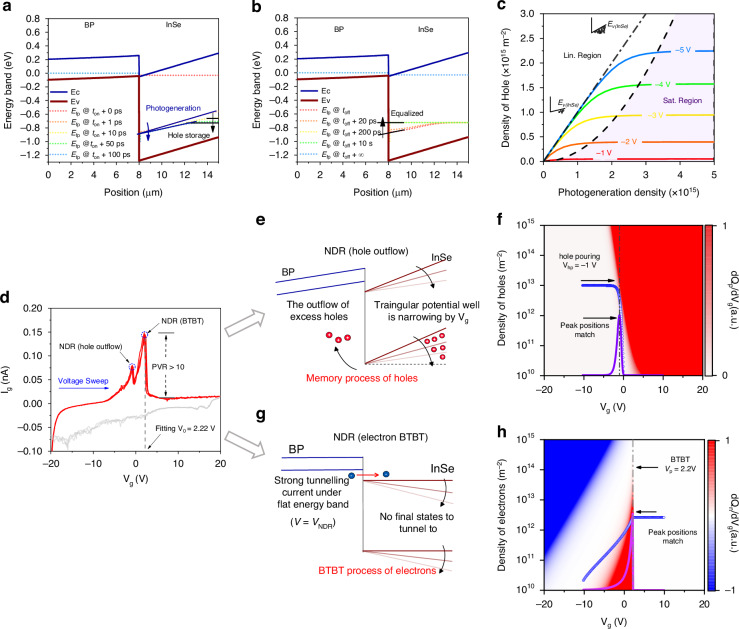
**Photomemory characteristics and dual NDR mechanism analysis of the critical BTBT memory. a** and **b** Transient behaviour of the hole quasi-Fermi levels near the rising and falling edges of a signal light pulse injected into the InSe/BP heterojunction energy bands. The signal light pulse is a square wave with a duration of 0.1 ns. The times of the rising and falling edges of the pulse are *t*_on_ and *t*_off_ = *t*_on_ + 0.1 ns, respectively. **c** Linear and saturation behaviour of the number density of stored holes in InSe with absorbed photogeneration density at *t*_off_ + 4 ms under different gate voltages *V*_g_ from −1 to −5 V. The signal light pulse is a square wave with a 1 ns duration. The dashed curve is the boundary between the linear and saturated regions. The dashed-dotted line shows the ideal curve when all the photogenerated holes are stored. **d** Experimental results show dual NDR in the hysteresis curve of the gate current when the gate voltage is swept from −20 to 20 V (red curve) and back (grey). The positive direction of the hysteresis current is defined as the direction from InSe to BP. The dashed line shows the flat band condition extracted from the saturation behaviour in (**c**). **e** The outflow of excess holes due to the decreasing potential well, resulting in an NDR in the hysteresis curves. **f** Calculated distribution of hole flow ${\rm{d}}{Q}_{{\rm{h}}}/{\rm{d}}V$ with gate voltage *V*_g_ and the total number of holes *Q*_h_ in InSe. **g** The tunnelling current is strongest when the energy band alignment is flat and decreases sharply when crossing over *V*_NDR._ **h** Calculated electron BTBT flow ${\rm{d}}{Q}_{{\rm{e}}}/{\rm{d}}V$ with gate voltage *V*_g_ and the total numb_e_r of electrons *Q*_e_ in InSe. Blue dots are time-dependent simulation results of t_h_e variation in *Q*_h_ and *Q*_e_, with the gate voltage *V*_g_ showing hole outflow near *V*_hp_ = −1 V and electron BTBT near *V*_BTBT_ = 2.2 *V*, respectively. Purple lines are the hole outflow current ${I}_{{\rm{h}}{\rm{p}}}=({\rm{d}}{Q}_{{\rm{h}}}/{\rm{d}}V)({\rm{d}}V/{\rm{d}}t)$ and electron BTBT current ${I}_{{\rm{B}}{\rm{T}}{\rm{B}}{\rm{T}}}=({\rm{d}}{Q}_{{\rm{e}}}/{\rm{d}}V)({\rm{d}}V/{\rm{d}}t)$. The time-dependent simulation results show that the positions of the peaks of the hole outflow current and BTBT current coincide with the two NDR peaks in (**d**)

The photoresponse of the critical BTBT memory can be described by the transient behaviour of the quasi-Fermi level and the temporal evolution of the BP/InSe/SiO_2_/Si structure (Fig. 3a, b and Supplementary Section 13, the fitting parameters are all illustrated in the “Method” section). In the ultrathin InSe layer, photoexcitation induces the quasi-Fermi level of holes to shift approximately parallel to the valence band. Simultaneously, under the modulation of *V*_g_, the holes redistribute inside InSe to reach a quasi-equilibrium, satisfying the Poisson equation. When the quasi-Fermi level is horizontal, the hole storage process is completed. Owing to the extremely short moving distance (nm) and high mobility in the InSe layer, the transition from the light injection state with a tilted quasi-Fermi level to the horizontal storage state occurs within 100 ps^42^, and prominent photomemory characteristics are achieved. Note that the curve of the density of holes with the photogeneration density (Fig. 3c) resembles the *I*_D_–*V*_D_ characteristics of a junction field-effect transistor (JFET); thus, the linear characteristics and accumulated effect can be explained using the JFET model. With increasing photogeneration density, the number of holes stored under different gate voltages almost coincides with the ideal linear curve until the pinch-off point, where a JFET transitions from the linear region to the saturation region, predicting a wide linearity range. When the saturation region is entered, the saturation value of hole storage is determined by the geometry of the triangular potential well formed by the InSe valence band and SiO_2_. The depth and width of the triangular potential well are equal to the potential drop in and thickness of the InSe layer, respectively, which coincide with the model of the saturation current for a JFET. ${V}_{{\rm{p}}}=2.2\,\text{V}$ can be derived from ${I}_{{\rm{D}}{\rm{S}}}/{I}_{{\rm{D}}{\rm{S}}{\rm{S}}}={(1-{V}_{{\rm{g}}}/{V}_{{\rm{P}}})}^{2}$ for a JFET when the energy band is horizontal, where ${I}_{{\rm{D}}{\rm{S}}}$ and ${I}_{{\rm{D}}{\rm{S}}{\rm{S}}}$ are the drain–source current and saturation current at zero gate–source voltage, respectively. It is noted that the thickness of InSe, which is related to the formation of the triangular potential well, significantly impacts the detection limitation.

The most direct evidence of BTBT is the NDR point in the *I*_d_*–V*_d_ curve in the horizontal direction in previous research; however, NDR in the horizontal direction cannot verify the detailed process in the vertical direction. In order to figure out the electron tunnelling in the memory process, we demonstrate the *I*_g_*–V*_g_ hysteresis curve and observe dual NDR points in Fig. 3d. The dual NDR points are located at ${V}_{{\rm{g}}}=-1\,\text{V}$ and ${V}_{{\rm{g}}}=2.2\,\text{V}$ in the forward voltage sweeping (Fig. 3d, Supplementary Sections 14, 15). The first NDR point represents the outflow of stored holes, as shown in Fig. 3e. When the voltage is sweeping, the holes are stored in the triangular potential well formed by InSe/SiO_2_. In contrast, the potential well is narrowing by decreasing *V*_g_, resulting in a current spike due to the excess hole outflow. As the diffusion current of holes varies with the gate voltage ${V}_{{\rm{g}}}$ and the density of holes ${Q}_{{\rm{p}}}$ in InSe, the detailed hole pouring process is demonstrated in Fig. 3f. Under any definite equivalent mobility ${\mathop{\mu }\limits^{ \sim }}_{{\rm{p}}}$, different ${Q}_{{\rm{p}}}$ values correspond to different peak positions, indicating that the memory-induced hole outflow process is history-dependent. The second NDR point represents the electron BTBT process, and the peak position is determined. When the energy band alignment reaches flat with the gate voltage sweeping, there is a strong tunnelling current at *V*_NDR_. When the gate voltage crosses over *V*_NDR_, the current decreases sharply for no final states to tunnel to, as shown in Fig. 3g, which is also consistent with the large PVR (peak valley ratio) in Fig. 3d. Similarly, the detailed electron tunnelling process is demonstrated in Fig. 3h, showing that the BTBT-induced NDR matches the experimental peak position of the NDR in Fig. 3d at ${V}_{{\rm{g}}}=2.2\,\text{V}$.

### Moving target tracking and recognition

A motion process can be regarded as image streaming across different frames, which can be memorized in optoelectronic memory devices. When combined with an interframe algorithm, the movement trajectory is exclusively defined^35,43^. The critical BTBT memory, with photomemory characteristics, exhibits the potential for motion detection. The optical characterization of critical BTBT memory is shown in Supplementary Section 16. The absorption and quantum efficiency are decisive factors in the application scope of the demonstrated device. Although the infinitesimal barrier facilitates the tunnelling process and exhibits high quantum efficiency, the limitation in absorption still requires further improvement.

**Fig. 4:**
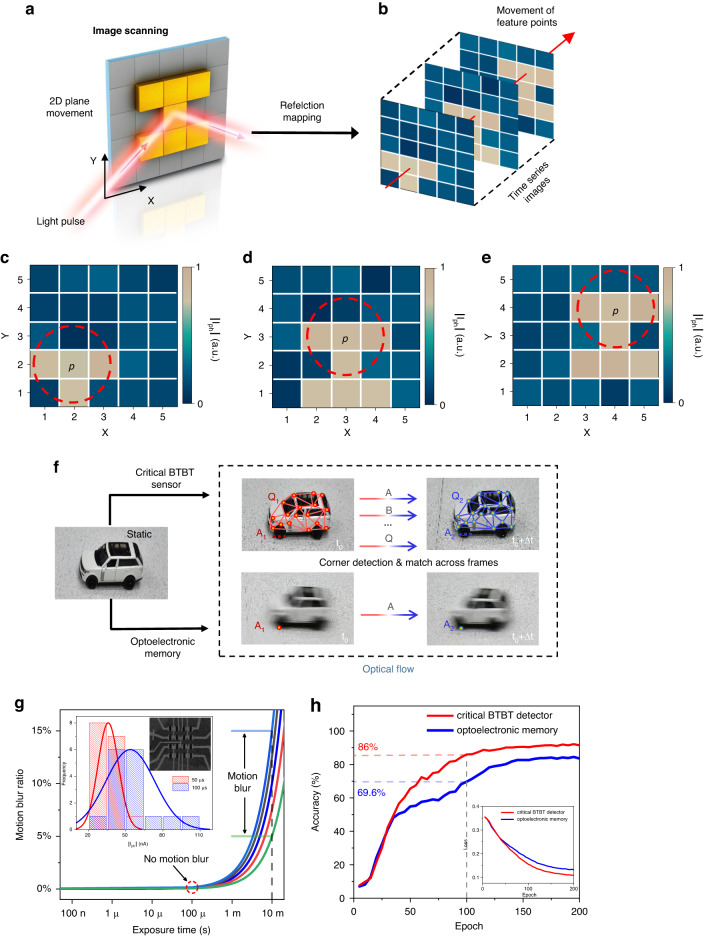
**Comparison between the critical BTBT memory and reported optoelectronic memories in terms of proof-of-concept moving target tracking and recognition. a** Reflective imaging using the customized “I”-shaped metal pattern, which can move in the 2D plane for image scanning. The laser pulse is 520 nm with a 200 μs duration. **b** The red arrow penetrates the same feature point across frames, representing its temporal movement. **c–e** Photocurrent mapping data scanned at different frames. *p* represents the same point of the customized “I”-shaped metal pattern, and the red dashed line contains feature point *p* and its neighbouring pixels. The feature point *p*, has the same movement trajectory as the whole, and target tracking is simplified to tracking the same points across frames, which is the basis of optical flow. **f** Double sets of moving trolley datasets obtained by the critical BTBT memory and other optoelectronic memories with an exposure time of 10 ms. The feature points in the two sets of time series images are detected by Harris corner detection and matched through the optical flow method. **g** The motion blur ratio is defined as the distance covered by the moving target during the exposure time divided by the length of the moving target. Relationship between the motion blur ratio and exposure time (also photomemory speed); different curves represent different trolley speeds. Motion blur exists when the exposure time is 10 ms, while almost no motion blur exists at 100 μs. Inset image: SEM view of the 4 × 4 critical BTBT memory and corresponding photomemory current in response to a 520 nm laser with different pulse widths, with red columns for 50 μs and blue for 100 μs. The solid lines are the results of fitting via a normal distribution. The preparation of arrays is shown in Supplementary Section 19. **h** The comparison of the accuracy between different neural networks, in which the training database is constructed by the critical BTBT memory and optoelectronic memories. Inset image: Loss function of the critical BTBT memory-based neural network and optoelectronic memory-based neural network

Figure 4a shows a schematic diagram of reflective image scanning in a proof-of-concept demonstration. A customized letter “I”-shaped metal pattern on a movable platform is utilized to simulate the moving target across frames (the schematic diagram of the imaging system is shown in Supplementary Section 17). The experimental imaging results in Fig. 4c–e show that even with a short exposure time, the information of the different frames is precisely memorized by our device and remains stable until the readout process. Figure 4f, based on the simulation results, presents the visualized results comparing the tracking ability of the critical BTBT memory and reported optoelectronic memory devices. The limited photomemory speed is set to 10 ms for comparison. To track the moving target, the feature points of frames at *t*_0_ are supposed to be detected by a Harris corner detector (Supplementary Section 18) and matched by optical flow across frames sequentially. For optoelectronic memory devices with a low memory speed, motion blur renders matching of feature points across frames difficult (only 1 feature point is matched). In contrast, for the critical BTBT memory, the clear and sharp profile contributes to the precise matching of 17 feature points, and the movement trajectory is exclusively distinguished. In addition, Fig. 4g presents the quantified results of the photomemory speed impact on motion blur. With a limited memory speed, the imaging results of the optoelectronic memory devices demonstrate varying degrees of motion blur. In contrast, for the proof-of-concept fabricated critical BTBT memory array, the 100 μs photomemory speed and exposure time guarantee a clear, sharp profile with motion blur eliminated. Figure 4h intuitively quantifies the influence of motion blur on the recognition accuracy of neural networks. At the 100th training epoch, the accuracy of our device-based neural network exceeds that of the optoelectronic memory-based neural network by more than 16% and finally stabilizes at 91.5%, proving a reliable motion recognition ability.

## Discussion

In conclusion, we have demonstrated a *critical BTBT-*based device with photomemory characteristics and a nearly infinitesimal barrier; under the elaborately designed triangular tunnelling region, recombination is suppressed, which leads to a sub-microsecond level response speed and non-volatile memory characteristics. The critical BTBT memory exhibits highly linear and NIR photomemory characteristics to cope with complex and dynamic environments. Furthermore, the dual NDR points observed in the hysteresis curve elucidate the memory and BTBT process, and the theoretical results are consistent with the experimental results. Finally, the comparison between the critical BTBT memory and optoelectronic memory also demonstrates that the photomemory speed (or exposure time) remarkably impacts the moving target tracking and recognition ability. In addition, considering future practical applications, while *critical BTBT memory* accelerates image processing, it also imposes higher demands on array uniformity. Significant device-to-device variability can degrade the accuracy of tracking and recognition. Therefore, reducing defective states and non-uniformities introduced during material growth and chip processing will be crucial for future research.

## Materials and methods

### Device fabrication

2D thin films are obtained by mechanical exfoliation from bulk materials supplied by HQ Graphene. A dry fixed-point transfer technology is utilized to transfer BP flakes onto InSe flakes. The substrate consists of highly p-doped silicon and 285 nm SiO_2_. The electrode patterns are fabricated by standard electron-beam photolithography (EBL). Then, after the thermal evaporation and lift-off processes, the source and drain electrodes, consisting of Cr/Au (10/30 nm), are deposited. After annealing in a nitrogen environment, the device is successfully fabricated.

### Characterization and measurements

A Keysight B1500A is utilized to measure the electronic characteristics of a Lake Shore probe station. All the operating environments are under a high vacuum (lower than 1 × 10^−3 ^Pa). Additionally, because both water and oxygen are involved in the degradation of BP, thus extending the oxidation area, double dehumidifiers are in operation during the whole experiment to maintain a dry environment. Laser pulses of 633, 830, and 940 nm are obtained with an ITC4001 Benchtop Laser Diode (Thor Labs). Sub-microsecond light pulses are first generated by a Rigol DG 5071 in the form of a digital electrical signal. Then, this signal controls a Coherent OBIS to generate 520 nm laser pulses. The laser performance is calibrated by a commercial Si-based photodetector, and the output signal is captured by a Tektronix MDO34 oscilloscope. The laser pulse is directed at the device via a reflective optical path. The device is located at the centre of the laser spot. Considering the protective layer doping effect^44^, there is no protective layer. In contrast, the operating environment is taken seriously to avoid degradation. The critical BTBT memory is preserved in a nitrogen glove box with low water and oxygen contents (both below 0.01 ppm). The long-term stability demonstration is shown in Supplementary Section 11.

### Imaging system and analysis

The customized “I”-shaped metal pattern is sequentially fabricated by EBL, thermal evaporation and lift-off processes, and Cr/Au (5/25 nm) is deposited onto the SiO_2_/Si substrate. The “I”-shaped pattern is placed on a mobile platform as a moving target. A 633 nm laser with a 200 μs pulse width is focused on the surface of the “I”-shaped pattern through an objective lens. After being reflected, the laser pulse irradiates the critical BTBT memory through an optical pathway consisting of an objective lens, a half mirror and a focusing lens. Finally, an Agilent 2902A semiconductor parameter analyser is utilized to perform time-dependent photocurrent measurements, and the current is read out after 0.5 s of optical stimulus. In this active, reflective imaging system, the scanning imaging principle is based on the different reflections of various substrates (Au and SiO_2_/Si substrates), resulting in different laser intensities. Through the negative photomemory current of the device at different positions, the shape of the moving target (“I”-shaped pattern) can be reconstructed.

### Numerical simulation of the photomemory performance and dual NDR

We adopt COMSOL Multiphysics to perform the finite element method (FEM) simulation of the energy band profile and temporal evolution of the BP/InSe/SiO_2_/Si structure. The material properties of SiO_2_ and Si are taken from the material library. The bandgaps^29^, affinities^29^, electron and hole mobilities^34,45–47^, electron and hole masses^48,49^, and dielectric constants^50,51^ of BP and InSe are taken from published articles and our KPFM measurement results (Fig. S4). The solution process includes a semiconductor equilibrium step solving only Poisson’s equation, a semiconductor stationary step, and a series of time-dependent steps with iteration parameters finely optimized. BTBT between the valence band of BP and the conduction band of InSe is realized by a customized generation and recombination process, with the explicit form of the current given in Supplementary Section 15. The calculations of the hole outflow current and BTBT current are based on Supplementary Section 14 and are performed using custom scripts written in MathWorks MATLAB. A MATLAB implementation of the Runge–Kutta method is adopted to perform the time-dependent simulation of the density of holes and electrons and the two corresponding NDR points in the hysteresis curve.

## Supplementary information

Supplementary information for Critical band-to-band-tunnelling based optoelectronic memory

## Data Availability

Data that support the findings of this study are available from the corresponding authors upon reasonable request.

## References

[1] Mennel, L. et al. Ultrafast machine vision with 2D material neural network image sensors. *Nature* **579**, 62–66, (2020). 32132692 10.1038/s41586-020-2038-x

[2] Tong, L. et al. 2D materials–based homogeneous transistor-memory architecture for neuromorphic hardware. *Science* **373**, 1353–1358, (2021). 34413170 10.1126/science.abg3161

[3] Wu, G. J. et al. Ferroelectric-defined reconfigurable homojunctions for in-memory sensing and computing. *Nat. Mater.* **22**, 1499–1506, (2023). 37770677 10.1038/s41563-023-01676-0

[4] Dodda, A. et al. Active pixel sensor matrix based on monolayer MoS2 phototransistor array. *Nat. Mater.* **21**, 1379–1387, (2022). 36396961 10.1038/s41563-022-01398-9

[5] Ali, M. A. et al. Graphene nanoparticles as data generating digital materials in industry 4.0. *Sci. Rep.* **13**, 4945, (2023). 36973318 10.1038/s41598-023-31672-y PMC10043272

[6] Wang, L. J. et al. Exciton-assisted electron tunnelling in van der Waals heterostructures. *Nat. Mater.* **22**, 1094–1099, (2023). 37365227 10.1038/s41563-023-01556-7 PMC10465355

[7] Li, W. J. et al. Quadrupolar-dipolar excitonic transition in a tunnel-coupled van der Waals heterotrilayer. *Nat. Mater.* **22**, 1478–1484, (2023). 37857887 10.1038/s41563-023-01667-1

[8] Spagnolo, M. et al. Experimental photonic quantum memristor. *Nat. Photonics* **16**, 318–323, (2022).

[9] Ning, H. K. et al. An in-memory computing architecture based on a duplex two-dimensional material structure for in situ machine learning. *Nat. Nanotechnol.* **18**, 493–500, (2023). 36941361 10.1038/s41565-023-01343-0

[10] Chen, H. W. et al. Logic gates based on neuristors made from two-dimensional materials. *Nat. Electron.* **4**, 399–404, (2021).

[11] Liao, F. Y. et al. Bioinspired in-sensor visual adaptation for accurate perception. *Nat. Electron.* **5**, 84–91, (2022).

[12] Yuan, S. F. et al. A wavelength-scale black phosphorus spectrometer. *Nat. Photonics* **15**, 601–607, (2021).

[13] Pi, L. J. et al. Broadband convolutional processing using band-alignment-tunable heterostructures. *Nat. Electron.* **5**, 248–254, (2022).

[14] Liu, K. Q. et al. An optoelectronic synapse based on α-In2Se3 with controllable temporal dynamics for multimode and multiscale reservoir computing. *Nat. Electron.* **5**, 761–773, (2022).

[15] Chen, R. X. et al. Breaking the temporal and frequency congestion of LiDAR by parallel chaos. *Nat. Photonics* **17**, 306–314, (2023).

[16] Lee, S. et al. Programmable black phosphorus image sensor for broadband optoelectronic edge computing. *Nat. Commun.* **13**, 1485, (2022). 35304489 10.1038/s41467-022-29171-1 PMC8933397

[17] Gadelha, A. C. et al. Gate-tunable non-volatile photomemory effect in MoS2 transistors. *2D Materials* **6**, 025036, (2019).

[18] Bai, S. L. et al. Nanographene-based heterojunctions for high-performance organic phototransistor memory devices. *Adv. Sci.* **10**, 2300057, (2023). 10.1002/advs.202300057 PMC10214218 36995051

[19] Zheng, H. Y. et al. Multichannel meta-imagers for accelerating machine vision. *Nat. Nanotechnol.* **19**, 471–478, (2024). 38177276 10.1038/s41565-023-01557-2 PMC11031328

[20] Zhou, G. D. et al. Full hardware implementation of neuromorphic visual system based on multimodal optoelectronic resistive memory arrays for versatile image processing. *Nat. Commun.* **14**, 8489, (2023). 38123562 10.1038/s41467-023-43944-2 PMC10733375

[21] Ashtiani, F., Geers, A. J. & Aflatouni, F. An on-chip photonic deep neural network for image classification. *Nature* **606**, 501–506, (2022). 35650432 10.1038/s41586-022-04714-0

[22] Chen, Y. T. et al. All-analog photoelectronic chip for high-speed vision tasks. *Nature* **623**, 48–57, (2023). 37880362 10.1038/s41586-023-06558-8 PMC10620079

[23] Zhou, F. C. & Chai, Y. Near-sensor and in-sensor computing. *Nat. Electron.* **3**, 664–671, (2020).

[24] Seo, S. Y. et al. Reconfigurable photo-induced doping of two-dimensional van der Waals semiconductors using different photon energies. *Nat. Electron.* **4**, 38–44, (2020).

[25] Cui, B. Y. et al. Ferroelectric photosensor network: an advanced hardware solution to real-time machine vision. *Nat. Commun.* **13**, 1707, (2022). 35361828 10.1038/s41467-022-29364-8 PMC8971381

[26] Wang, T. Y. et al. Image sensing with multilayer nonlinear optical neural networks. *Nat. Photonics* **17**, 408–415, (2023).

[27] Wang, Y. et al. Optoelectronic synaptic devices for neuromorphic computing. *Adv. Intell. Syst.* **3**, 2000099, (2021).

[28] He, T. et al. On-chip optoelectronic logic gates operating in the telecom band. *Nat. Photonics* **18**, 60–67, (2024).

[29] Lv, Q. S. et al. Interlayer band-to-band tunneling and negative differential resistance in van der Waals BP/InSe field-effect transistors. *Adv. Funct. Mater.* **30**, 1910713, (2020).

[30] Wu, F. et al. AsP/InSe van der Waals tunneling heterojunctions with ultrahigh reverse rectification ratio and high photosensitivity. *Adv. Funct. Mater.* **29**, 1900314, (2019).

[31] Xiong, X. et al. A transverse tunnelling field-effect transistor made from a van der Waals heterostructure. *Nat. Electron.* **3**, 106–112, (2020).

[32] Kim, S. et al. Thickness-controlled black phosphorus tunnel field-effect transistor for low-power switches. *Nat. Nanotechnol.* **15**, 203–206, (2020). 31988502 10.1038/s41565-019-0623-7

[33] Zhou, F. C. et al. Optoelectronic resistive random access memory for neuromorphic vision sensors. *Nat. Nanotechnol.* **14**, 776–782, (2019). 31308498 10.1038/s41565-019-0501-3

[34] Sun, C. et al. Ab initio study of carrier mobility of few-layer InSe. *Appl. Phys. Express* **9**, 035203, (2016).

[35] Zhang, Z. H. et al. All-in-one two-dimensional retinomorphic hardware device for motion detection and recognition. *Nat. Nanotechnol.* **17**, 27–32, (2022). 34750561 10.1038/s41565-021-01003-1

[36] Syed, G. S. et al. Atomically thin optomemristive feedback neurons. *Nat. Nanotechnol.* **18**, 1036–1043, (2023). 37142710 10.1038/s41565-023-01391-6

[37] Gao, A. Y. et al. Observation of ballistic avalanche phenomena in nanoscale vertical InSe/BP heterostructures. *Nat. Nanotechnol.* **14**, 217–222, (2019). 30664752 10.1038/s41565-018-0348-z

[38] Miao, J. S. et al. Heterojunction tunnel triodes based on two-dimensional metal selenide and three-dimensional silicon. *Nat. Electron.* **5**, 744–751, (2022).

[39] Zhao, S. W. et al. Highly polarized and fast photoresponse of black phosphorus-InSe vertical p–n heterojunctions. *Adv. Funct. Mater.* **28**, 1802011, (2018).

[40] Wang, H. D. et al. Repression of interlayer recombination by graphene generates a sensitive nanostructured 2D vdW heterostructure based photodetector. *Adv. Sci.* **8**, 2100503, (2021). 10.1002/advs.202100503 PMC8336618 34014610

[41] Zubair, M. et al. Gate-tunable van der Waals photodiodes with an ultrahigh peak-to-valley current ratio. *Small* **19**, 2300010, (2023). 10.1002/smll.202300010 37058131

[42] Li, W. B., Poncé, S. & Giustino, F. Dimensional crossover in the carrier mobility of two-dimensional semiconductors: the case of InSe. *Nano Lett.* **19**, 1774–1781, (2019). 30734566 10.1021/acs.nanolett.8b04799

[43] Ölveczky, B. P., Baccus, S. A. & Meister, M. Segregation of object and background motion in the retina. *Nature* **423**, 401–408, (2003). 12754524 10.1038/nature01652

[44] Wang, C. Y. et al. Gate-tunable van der Waals heterostructure for reconfigurable neural network vision sensor. *Sci. Adv.* **6**, eaba6173, (2020). 32637614 10.1126/sciadv.aba6173 PMC7314516

[45] Ling, X. et al. The renaissance of black phosphorus. *Proc. Natl Acad. Sci. USA* **112**, 4523–4530, (2015). 25820173 10.1073/pnas.1416581112 PMC4403146

[46] Youngblood, N, et al. Waveguide-integrated black phosphorus photodetector with high responsivity and low dark current. *Nat. Photonics* **9**, 247–252, (2015).

[47] Zhou, L. L. et al. Electrical and optical properties of InSe with various interfaces. *Appl. Phys. Lett.* **121**, 071603, (2022).

[48] Low, T. et al. Tunable optical properties of multilayer black phosphorus thin films. *Phys. Rev. B* **90**, 075434, (2014).

[49] Şahin, H. et al. Monolayer honeycomb structures of group-IV elements and III–V binary compounds: first-principles calculations. *Phys. Rev. B* **80**, 155453, (2009).

[50] Zhou, H. B. et al. Unusual phonon behavior and ultra-low thermal conductance of monolayer InSe. *Nanoscale* **10**, 480–487, (2018). 10.1039/c7nr07779c 29231225

[51] Morita, A. Semiconducting black phosphorus. *Appl. Phys. A* **39**, 227–242, (1986).

