# Supplementary material for: Critical band-to-band-tunnelling based optoelectronic memory
Source: Light Sci Appl. 2025 Feb 7;14:72. doi: 10.1038/s41377-025-01756-7 (PMC11802729; doi:10.1038/s41377-025-01756-7)
Supplement: Supplementary file 1 — Supplementary information for Critical band-to-band-tunnelling based optoelectronic memory [file 41377_2025_1756_MOESM1_ESM.docx]

Supplementary information for

**Critical band-to-band-tunnelling based optoelectronic memory**

Hangyu Xu^1,2,§^, Runzhang Xie^1,§^, Jinshui Miao^1,2,§^, Zhenhan Zhang^1,3,§^, Haonan Ge^1^, Xuming Shi^4^, Min Luo^1,2^, Jinjin Wang^1,2^, Tangxin Li^1,2^, Xiao Fu^1^, Johnny C. Ho^5^, Peng Zhou^3^, Fang Wang^1,2*^, Weida Hu^1,2*^

^1^State Key Laboratory of Infrared Physics, Shanghai Institute of Technical Physics, Chinese Academy of Sciences, 500 Yu Tian Road, Shanghai 200083, China

^2^University of Chinese Academy of Sciences, Beijing, China

^3^ASIC & System State Key Laboratory, School of Microelectronics, Fudan University, Shanghai, China

^4^Shanghai Research Institute for Intelligent Autonomous Systems, Tongji University, Shanghai, 200092, China

^5^Department of Materials Science and Engineering and State Key Laboratory of Terahertz and Millimeter waves, City University of Hong Kong, Kowloon Tong, Hong Kong SAR, China

^§^These authors contributed equally: Hangyu Xu, Runzhang Xie, Jinshui Miao and Zhenhan Zhang

^*^Correspondence should be addressed to: [wdhu@mail.sitp.ac.cn](mailto:wdhu@mail.sitp.ac.cn); [fwang@mail.sitp.ac.cn](mailto:fwang@mail.sitp.ac.cn)

**Table of contents**

**Section 1 | Comparison of photodetectors and critical BTBT memory in image processing system..3**

**Section 2 | Material characteristics of critical BTBT memory………………………………………....…...4**

**Section 3 | The electrical characterization of individual transistors and PN junctions………………..7**

**Section 4 | Equivalent circuit of BP/InSe/SiO_2_/Si and related structures and its RC time constant...8**

**Section 5 | The memory window of other devices……………………………………………..…………...13**

**Section 6 | Impact of BTBT on photomemory characteristics…………………………………….……...15**

**Section 7 | Comparing performances of similar structures…………………………..……………...…...16**

**Section 8 | Positive photomemory current of critical BTBT-based semi-floating gate device……...22**

**Section 9 |** **Laser pulse characterization………………………………………….…………..……….……...23**

**Section 10 | Negative photomemory characteristics of critical BTBT memory……………………….24**

**Section 11 | Time stability of critical BTBT memory…………….…………………………….……..……..26**

**Section 12 |** **The robustness of critical BTBT memory………………………………………………..……27**

**Section 13 |** **Analytic model of InSe/BP heterojunction energy band……………………………..….....28**

**Section 14 |** **Hole diffusion current of InSe/BP heterojunction……………………………..………….....30**

**Section 15 |** **Band-to-band tunnelling between InSe and BP…………………………………………......31**

**Section 16 |** **Optical characterization of critical BTBT memory………………………………………….35**

**Section 17 |** **Schematic diagram of the reflection imaging system……………………………..……….38**

**Section 18 |** **Evaluation of tracking ability……………………………………………………………..…..…39**

**Section 19 |** **Critical BTBT memory array demonstration…….…………………….………………..…….40**

**Section 20 |** **Benchmark of the optoelectronic memories………….…………………….……………….42**

**Section 1. Comparison of photodetectors and critical BTBT memory in image processing system**

Memristors are experts in processing neural network tasks, greatly reducing power consumption through analogue computing. For traditional photodetectors, in the memristor-based image processing system, the photocurrent during one exposure time is supposed to pass through the integrator circuit and be memorized in binary memory after A/D conversion for later processing. Then, the data in binary memory will be converted into analogue information (by using another D/A conversion) under clock signal control and input into the memristor array. In the entire system, A/D conversion is the main power-consuming process (each is about milliwatts). Our approach with critical BTBT memory arrays, combining sensing, integrating and memory, optimizes the image processing system through the *“fully analogue pathway”.* During exposure, the current will continuously increase (or decrease) and hold still even without light, meaning that the time series images are “memorized” in the arrays. Under the control of a digital clock signal, critical BTBT memory arrays can input the image data without additional A/D conversion.

**
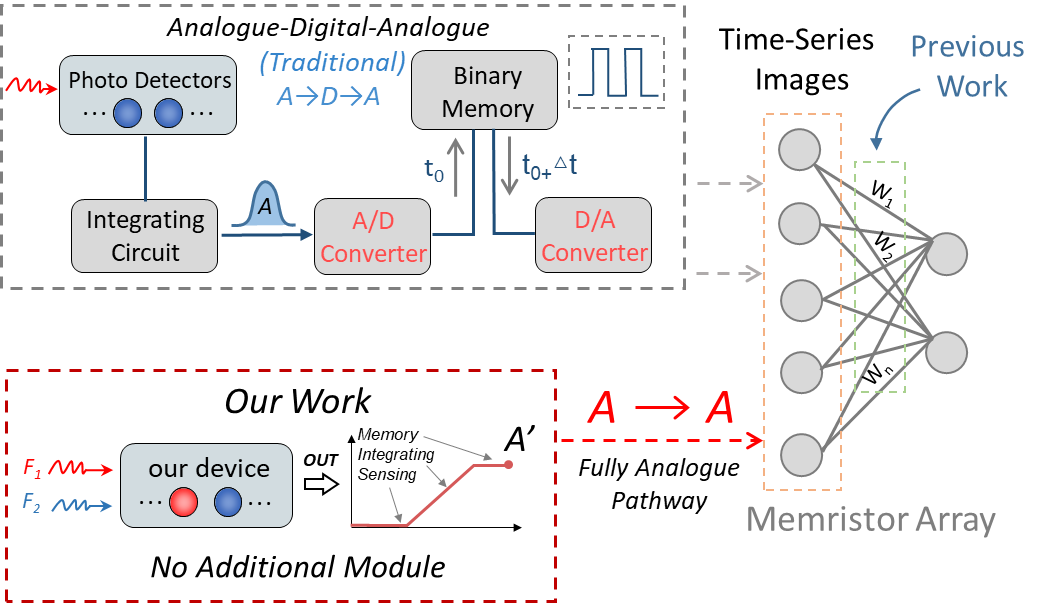
**

**Fig. S1 | The difference between our device and photodetectors in the memristor-based image processing system.** For a traditional photodetector, the analogue information is successively converted into digital and analogue through two converters. For our approach, critical BTBT memory establishes a *“fully analogue pathway”* between sensing and computing, which means that the information will transfer efficiently without any redundant conversion.

**Section 2. Material characteristics of critical BTBT memory**

**Ⅰ. Thickness and energy band alignments of critical BTBT memory by AFM and KPFM**

**
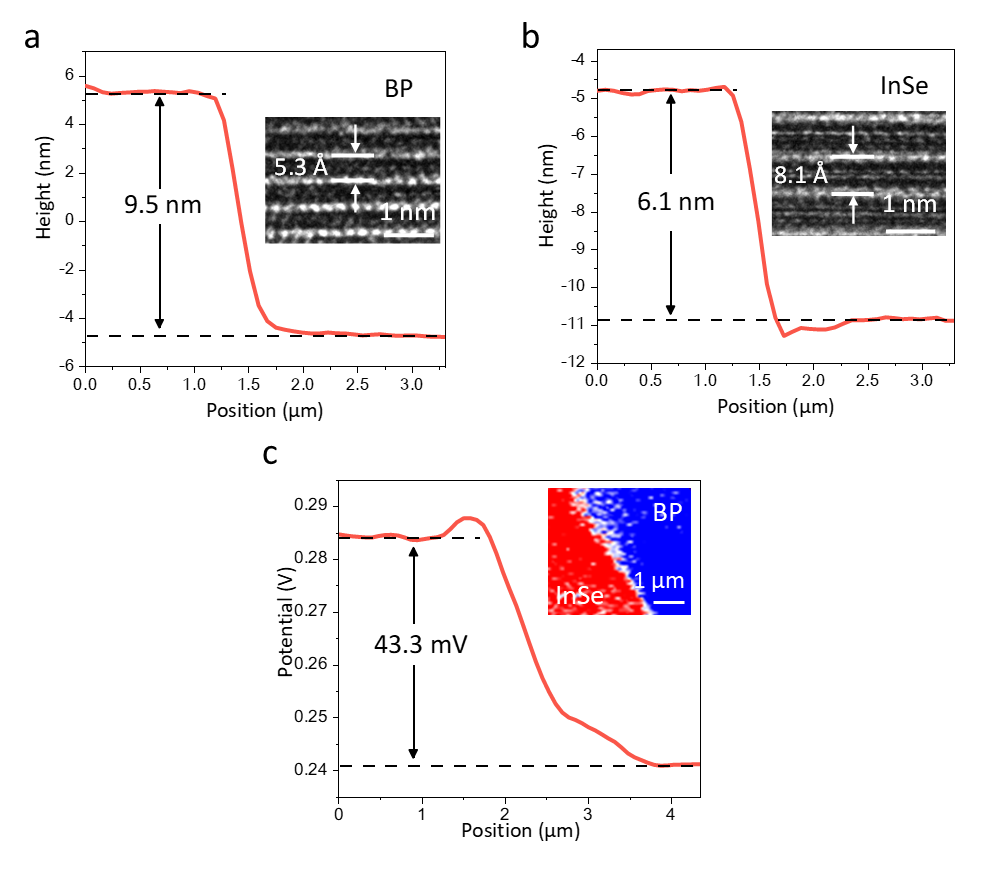
**

**Fig. S2 | Thickness and contact potential difference characterization of critical BTBT memory. a-b,** Height of BP, InSe thin films, respectively. The corresponding thickness is 9.5 nm and 6.1 nm, respectively. According to TEM results, the BP thin film has about 19 layers, and the InSe thin film has about 7 layers. **c,** Contact potential difference between BP and InSe thin films. Inset: KPFM image of BP and InSe thin films.

**Ⅱ. The typical Raman spectra of individual layers and overlapping regions**

Raman spectra of the overlapped region and individual layers, respectively, are consistent with the previous work^1,2^.

**
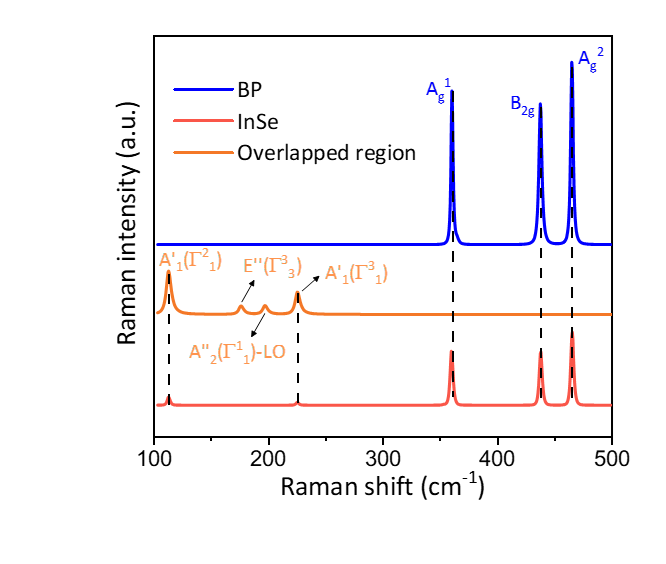
**

**Fig. S3 | Raman spectra characterization.** Raman spectra of isolated BP (blue), InSe flakes (red) and their overlapped region (orange). The typical Raman characteristic peaks of BP are located at 360.4 (A_g_^1^), 437.6 (B_2g_) and 465 cm^-1^ (A_g_^2^), respectively. The typical Raman characteristic peaks of individual InSe are located at 113 (A’_1_(Γ^2^_1_)), 176.5 (E’’(Γ^3^_3_), 197.3 (A’’_2_(Γ^1^_1_)-LO), 225.7 (A’_1_(Γ^3^_1_). The overlapped region’s five typical Raman peaks are consistent with the individuals' peaks, indicating a high quality of the transferred materials.

**Ⅲ. Element characterization of critical BTBT memory.**

Before the energy dispersive X-ray spectroscopy (EDS) measurement, this device is exposed to the same operating and preservation environment mentioned in the method for 7 days.

**
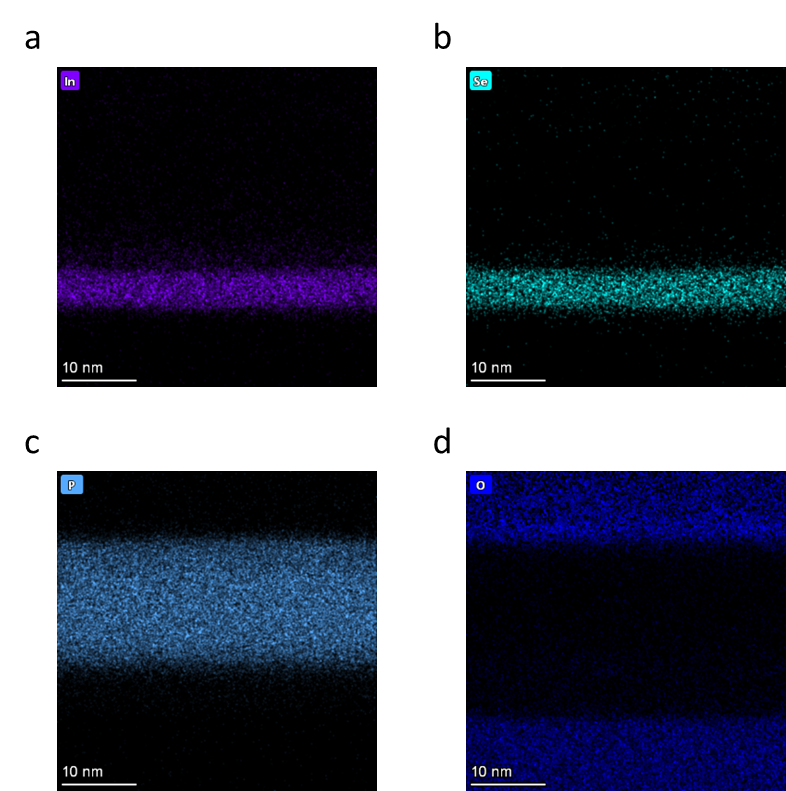
**

**Fig. S4 | The element characterization of critical BTBT memory by EDS. a-d,** The distribution of In, Se, P, and O, respectively. No oxygen element distribution is at the interface, suggesting a clean and sharp interface.

**Section 3. The electrical characterization of individual transistors and PN junctions**

**Ⅰ. The electrical characterization of BP and InSe transistors**

The output characteristics curves of BP and InSe are shown below. InSe has a Schottky contact with an ultimate low current at 10^-12^ A, which reaches the limitation of the experimental instrument. In contrast, BP exhibits an Ohmic contact with no memory window.

**
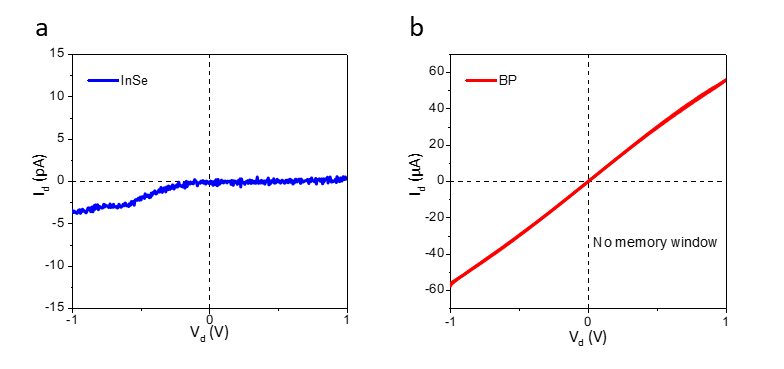
**

**Fig. S5 | *I_d_-V_d_* characteristics of BP and InSe transistors. a,** Linear scale *I_d_-V_d_* output characteristics of InSe transistor. **b,** Linear scale *I_d_-V_d_* output characteristics of BP transistor.

**Ⅱ. The electrical characterization of InSe-based PN junctions**

The output characteristics curves of BP/InSe, MoTe_2_/InSe and WSe_2_/InSe heterojunctions are shown below, respectively (InSe is the source). Considering that only under a negative back gate voltage leads to tunnelling to an available final state, the output characteristics of InSe-based PN junctions are demonstrated at positive bias. All of the PN junction currents are ultimately low and reach the limitation of the experimental instrument, illustrating a generalized interface carrier blocking effect^1,3-7^ in the InSe-based PN junctions.


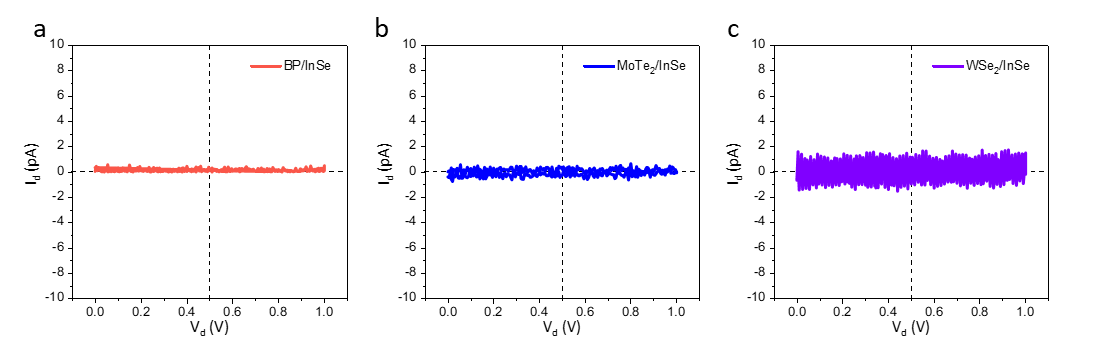


**Fig. S6 | *I_d_-V_d_* characteristics of InSe-based PN junctions. a,b,c,** Linear scale *I_d_-V_d_* output characteristics of BP/InSe, MoTe_2_/InSe and WSe_2_/InSe, respectively.

**Section 4. Equivalent circuit of BP/InSe****/SiO_2_/Si and related structures and its RC time constant**

In this section, we will establish the equivalent circuit of the channel/InSe/SiO_2_/Si structure, analyze the relationship between the hysteresis curve and key parameters of the equivalent circuit, and further discuss the influence of the leakage current of SiO_2_ on the hysteresis curve in the channel/InSe/SiO_2_/Si structure.

**Ⅰ. The equivalent circuit of BP/InSe/SiO_2_/Si**

As is presented in **Section. 13**, the interface of InSe/SiO_2_ and the interface of SiO_2_/Si forms a capacitor $C_{\text{storage}}$ leading to the major part of the gate voltage drop. (**Fig. S7a**) When a negative gate voltage is applied, the InSe side of the InSe/SiO_2_ interface of the capacitor $C_{\text{storage}}$ may store light-injected holes or electrons. The experimental results in **Section. 3** shows that the interface between InSe and the channel material (BP, MoTe_2_, etc.) has a strong interface-blocking effect on carriers. In the equivalent circuit in this section, this blocking effect is equivalent to a resistor $R_{\text{interface}}$.


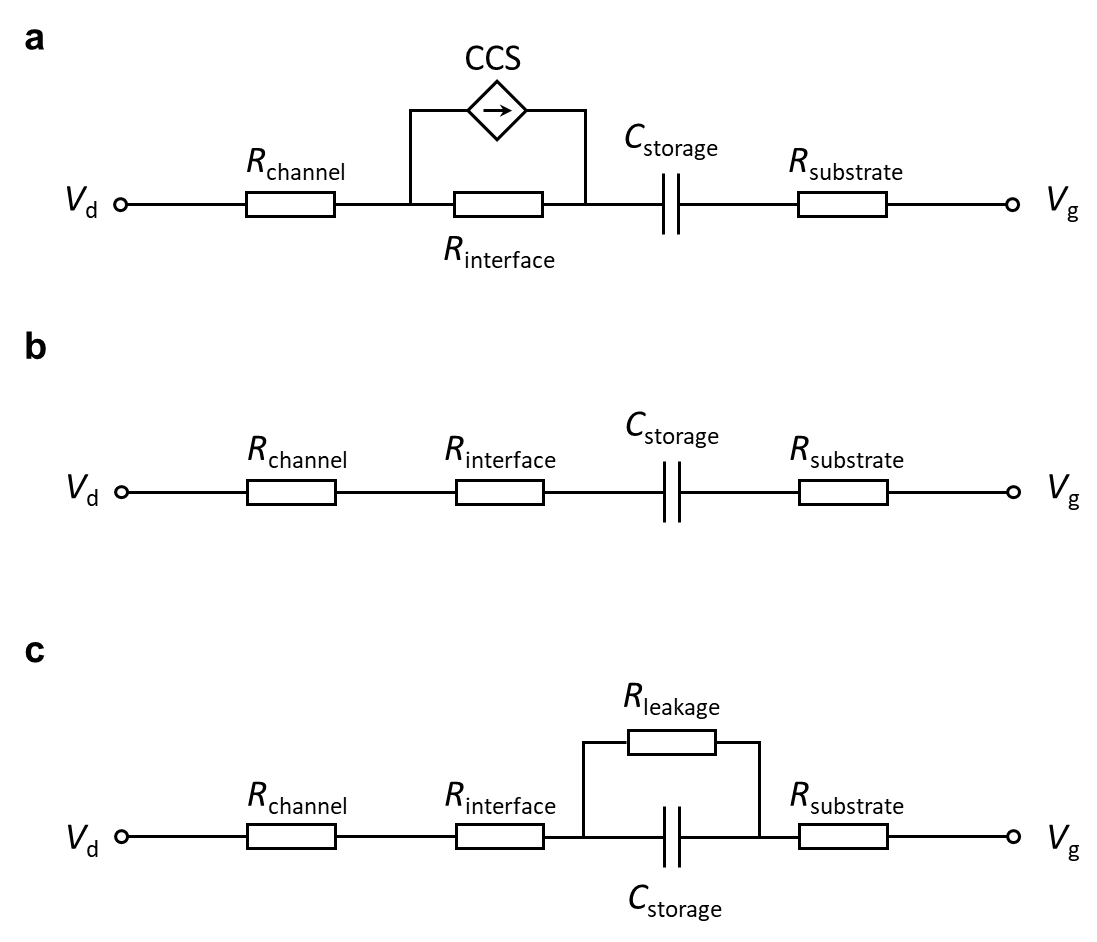


**Fig. S7 | The equivalent circuit of channel/InSe/SiO_2_/Si structure.** **a,** The equivalent circuit of BP/InSe/SiO_2_/Si structure with critical band-to-band tunnelling across the BP/InSe interface. **b,** The equivalent circuit of MoTe_2_/InSe/SiO_2_/Si or WSe_2_/InSe/SiO_2_/Si structure, or equivalent circuit of BP/InSe/SiO_2_/Si structure neglecting the critical band-to-band tunnelling current. **c,** Equivalent circuit with SiO_2_ leakage current.

**Ⅱ. RC time constant**

The band-to-band tunnelling current at the BP/InSe interface is not only affected by the current value of the gate voltage but also related to the gate voltage and transient current experienced in the past. In the equivalent circuit in this section, without loss of generality, we approximate it as a controlled current source (CCS) controlled by the external environment and states in the past. The discussion on the band-to-band tunnelling current is elaborated in the **Section. 15**, and this section concentrates on the equivalent circuit and the influence of its key parameters on the hysteresis curve. Noticing that the experiment result of *I_d_-V_g_* curves shows no observable influence of band-to-band tunnelling (*I_g_* current is more sensitive than *I_d_* current in analysis of the charge stored in gate), the equivalent circuit of the MoTe_2_/InSe/SiO_2_/Si structure should be the same as that of the BP/InSe/SiO_2_/Si structure, we will proceed with the subsequent discussion on memory window in this section based on the MoTe_2_/InSe/SiO_2_/Si structure (**Fig. S7b**). The consistency of the hysteresis curve behaviour of BP/InSe/SiO_2_/Si and MoTe_2_/InSe/SiO_2_/Si also supports this analysis. Considering that the junction capacitance of the PN junction mainly affects the high-frequency characteristics of the device, in the equivalent circuit for hysteresis curve measurement within the time scale of tens of milliseconds, we will neglect the junction capacitance $C_{\text{junction}}$ at the channel/InSe interface. The channel layer and the substrate also have small resistances. Compared with the resistance $R_{\text{interface}}$, these two resistances are small in the BP/InSe/SiO_2_/Si, MoTe_2_/InSe/SiO_2_/Si structure, and similar structures, but in the BP/SiO_2_/Si structure without interface carrier blocking effect, they play a dominant role. In this equivalent circuit, they are equivalent to resistance $R_{\text{channel}}$ and resistance $R_{\text{substrate}}$, respectively. As can be seen from **Fig. S7b**, this equivalent circuit is an RC series circuit with RC time constant

$\tau_{RC}=C_{\text{storage}}\left(R_{\text{interface}}+R_{\text{channel}}+R_{\text{substrate}}\right)$.

Since both the characteristic time of the writing process and the characteristic time of the discharging process are the RC time constant $\tau_{RC}$ of the circuit, devices with invariant RC time constants are not suitable for memory with the electric writing and reading process. DRAM is a typical case of modulating the equivalent RC time constant $\tau_{RC}$ of the circuit by changing the channel resistance of the logic gate to achieve storage. (**Fig. S8**) If the carrier is directly injected into the memory capacitor by light excitation, the problem of low-speed electrical writing caused by the large RC time constant $\tau_{RC}$ can be avoided. However, light injection will simultaneously inject the same number of electrons and holes so that the light-excited carriers can not only flow through the resistor but also be lost by recombining electron-hole pairs. Based on the *critical band-to-band tunnelling* mechanism, one type of carrier in the BP/InSe/SiO_2_/Si structure is quickly expelled, which can prevent the recombination process from occurring. The discussion of the *critical band-to-band tunnelling* mechanism is left to the **Section. 16**. As another mechanism affecting the memory performance, the RC transient response (including the RC time constant) of the channel/InSe/SiO_2_/Si structure can be analyzed clearly through the hysteresis curve.


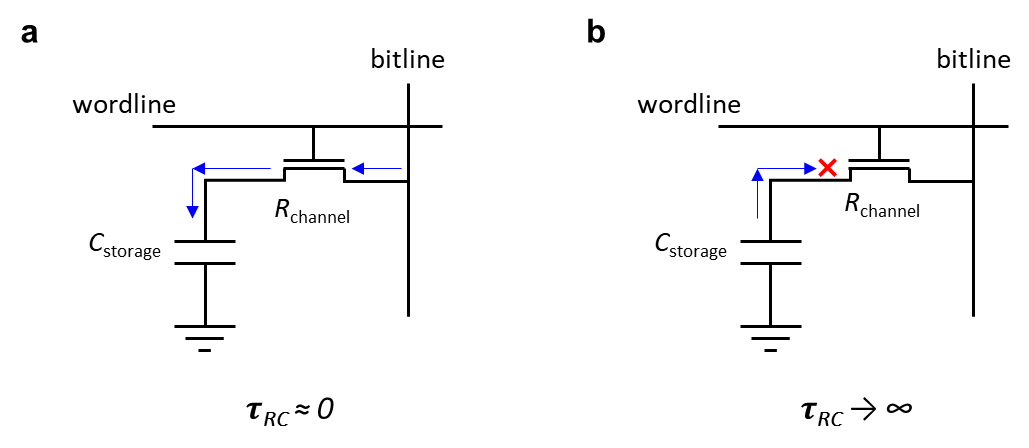


**Fig. S8 | Schematic of DRAM working principle.** **a,** Writing process of the DRAM cell. During the writing process, the channel of the DRAM is opened to obtain a small resistance $R_{\text{channel}}$ and an RC time constant $\tau_{RC}$ so that carriers can charge the capacitor $C_{\text{storage}}$ through the channel to achieve high-speed writing. **b,** The memory state of the DRAM cell. When the DRAM cell is in the memory state, the channel of DRAM is closed, making its RC time constant $\tau_{RC}$ much larger to prevent the stored charge from flowing away.

**Ⅲ. Transient simulation of equivalent circuits**

The transient performance of the equivalent circuit is simulated by PSPICE, and the results are shown in **Supplementary Fig.9**. For a given period *T* of the hysteresis curve measurement, the simulation results show the cases that the RC time constant of the equivalent circuit is much smaller, approximately equal to, and much larger than the period *T* of the measurement. In the case $\tau_{\text{RC}}\ll T$, the charging and discharging speed of the RC circuit can keep up with the changing speed of the gate voltage, and the influence of the historical state on the circuit can be ignored. The circuit does not have any storage effect when $\tau_{\text{RC}}\ll T$, because any stored charge will be lost at a speed faster than *T*. This situation is consistent with the measured results of the hysteresis curve of the BP/SiO_2_/Si structure. Since no blocking effect on carriers at the interface is provided, the RC time constant of the equivalent circuit is extremely small. The measurement and simulation results show that, in this case, the $I_{d}-V_{g}$ curve has nearly no hysteresis, as the BP/SiO_2_/Si curve in **Fig. 1b** in the main text and **Fig. S9a-d** below.


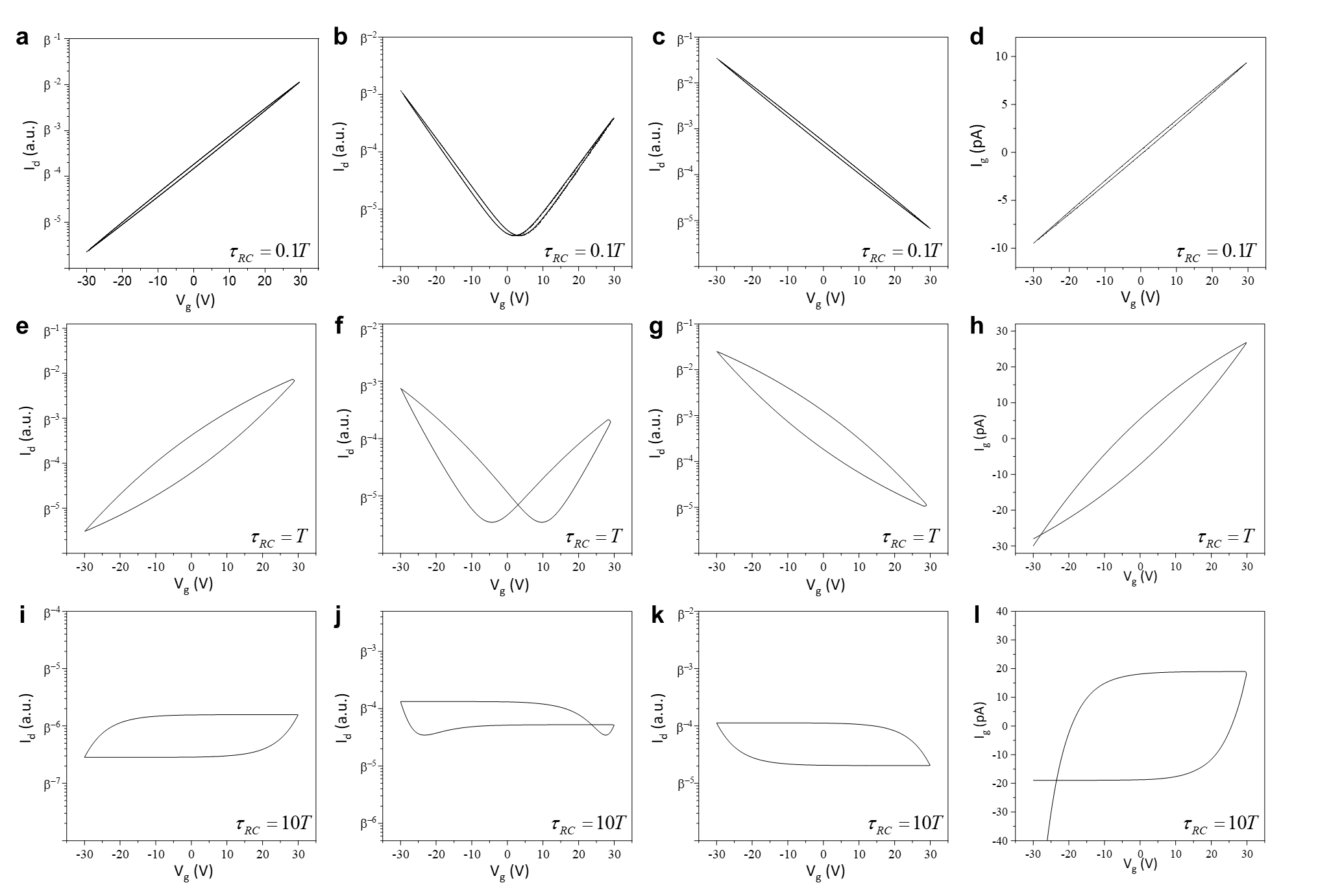


**Fig. S9 | PSPICE transient simulation results of equivalent circuits with different types of channel materials and different RC time constants.** Fixing the period of simulation time *T*, $I_{d}-V_{g}$ and $I_{g}-V_{g}$ curves for the equivalent circuit when the RC time constant is 0.1 *T* (**a-d**), *T* (**e-h**), and 10 *T* (**i-l**) for n-type (**a, e, i**), bipolar (**b, f, j**), and p-type channel (**c, g, k**), respectively. $\beta=\exp\left(-qb/k_{B}T\right)$ is a phenomenological parameter about the average potential drop in the channel selected independently to demonstrate the trend of $I_{d}-V_{g}$ curves in different cases. *k_B_*, *q*, and *T* = 300 K are the Boltzmann constant, elementary charge, and the working temperature, respectively. $I_{d}-V_{g}$ curves are recorded after multiple periods of scanning. $I_{g}-V_{g}$ curves show the case of the first period of scanning.

In the case $\tau_{\text{RC}}\sim T$, the RC equivalent circuit gradually becomes unable to keep up with the changing speed of the gate voltage, and the hysteresis becomes significant. However, this device still does not have a memory ability on the time scale of *T* because the current in the charging and discharging process is still jointly affected by the gate voltage and the historical state, as is shown in **Fig. S9e-h**. In the case $\tau_{\text{RC}}\gg T$, the charging and discharging process of the channel/InSe/SiO_2_/Si structure cannot keep up with the change of the external gate voltage at all. This shows that it is difficult for the charge outside the device to enter the storage capacitor $C_{\text{storage}}$, and it is also difficult for the charge in the storage capacitor $C_{\text{storage}}$ to escape, as the BP/InSe/SiO_2_/Si curve in **Fig. 1b** shown in the main text. Thus, this phenomenon was found in multiple channel/InSe/SiO_2_/Si structures containing InSe because of the carrier-blocking effect of the channel/InSe interface. However, unless a mechanism allows one type of carrier to leave $C_{\text{storage}}$ quickly, like critical band-to-band tunnelling, the photogenerated electrons and photogenerated holes will eventually be recombined, and memory ability will be lost.

**Section 5. The memory window of other devices**

Note that InSe, due to the interface blocking effect, definitely increase the RC time constant in the transfer characteristic curves test, rendering an enlarged memory window. As shown in **Fig. S10**, the memory window of different semiconductors on InSe has enlarged compared with the corresponding individual transistors. However, only for AsP on InSe, the negative photomemory current exists.

**Ⅰ. Memory window of WSe_2_ (on InSe) and MoTe_2_ (on InSe)**

**
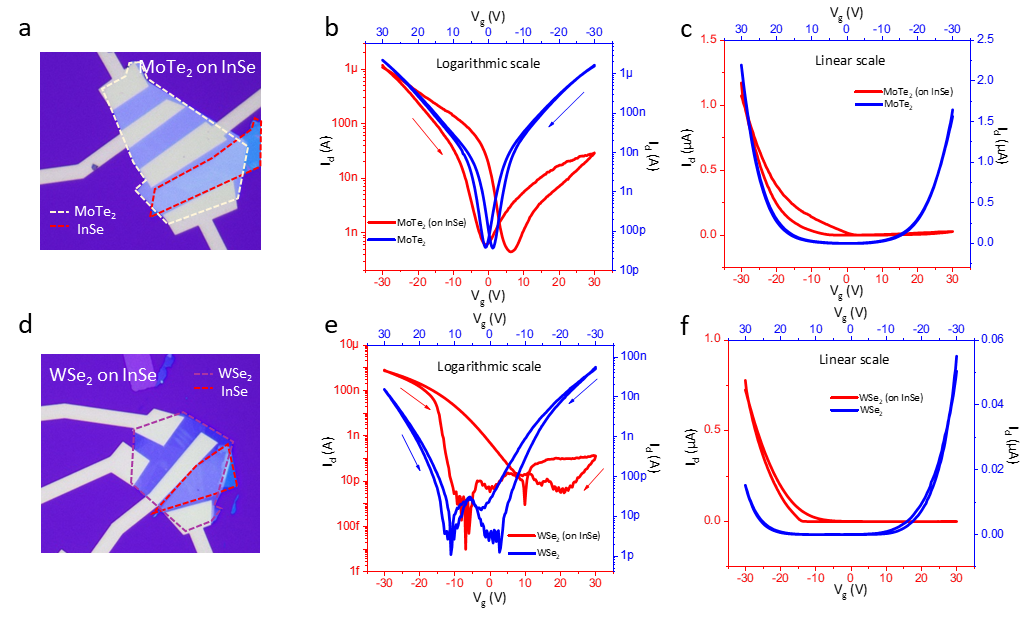
**

**Fig. S10 | Transfer characteristic curves of WSe_2_ (on InSe) and MoTe_2_ (on InSe).** **a,** Optical photograph of MoTe_2_ on InSe device. **b,** Transfer characteristic curves of MoTe_2_ (blue line) and MoTe_2_ on InSe (red line) in logarithmic scale. **c,** Transfer characteristic curves of MoTe_2_ (blue line) and MoTe_2_ on InSe (red line) in linear scale. **d,** Optical photograph of WSe_2_ on InSe device. **e,** Transfer characteristic curves of WSe_2_ (blue line) and WSe_2_ on InSe (red line) in logarithmic scale. **f,** Transfer characteristic curves of WSe_2_ (blue line) and WSe_2_ on InSe (red line) in linear scale.

**Ⅱ. Memory window of AsP (on InSe)**

**
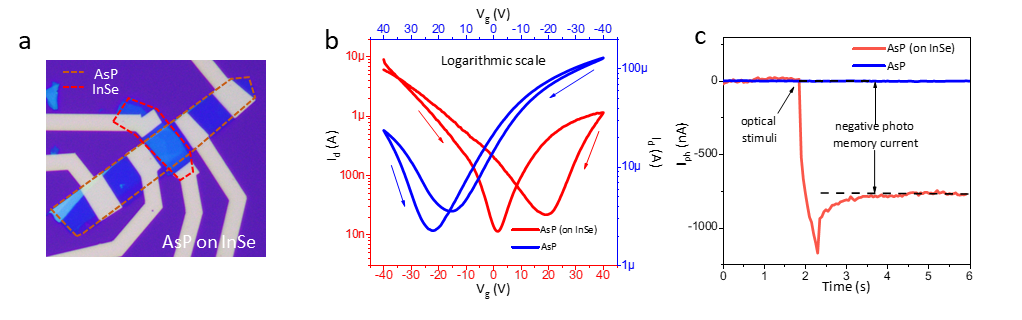
**

**Fig. S11 | Transfer characteristic curves of AsP (on InSe).** **a,** Optical photograph of AsP on InSe device. **b,** Transfer characteristic curves of AsP (blue line) and AsP on InSe (red line) in logarithmic scale. **c,** The photomemory performance of AsP on InSe device.

**Ⅲ. *I_g_-V_g_* hysteresis curve of MoTe_2_ (on InSe) and WSe_2_ (on InSe)**

The corresponding *I_g_-V_g_* characterization of MoTe_2_ on the InSe device and WSe_2_ on the InSe device in hysteresis curves.

**
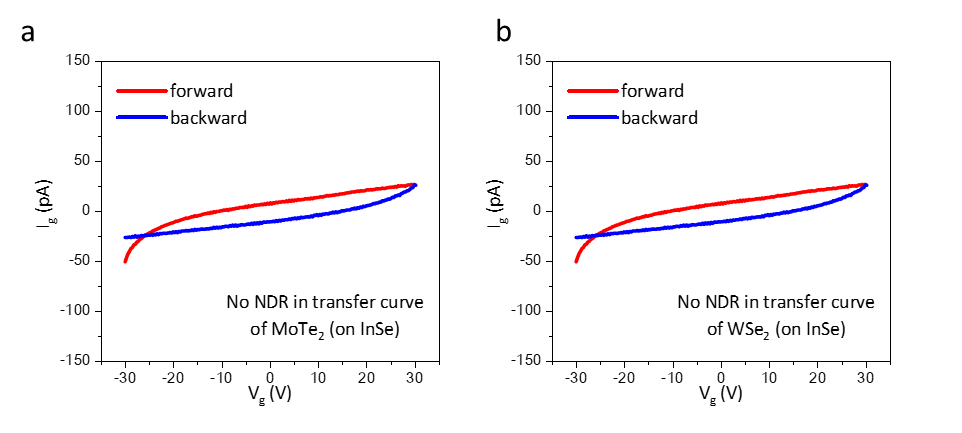
**

**Fig. S12 | *I_g_-V_g_* characterization of MoTe_2_ on InSe and WSe_2_ on InSe.** Red lines represent the voltage forward sweeping direction, while blue lines represent the voltage backwards sweeping direction. There is no NDR point in either device's *I_g_-V_g_* hysteresis curves.

**Section 6. Impact of BTBT on photomemory characteristics**

In a 940 nm laser stimuli cycle, the falling time is significantly longer than the rising time, indicating volatile negative photomemory characteristics. When the laser stimulus is 1310 nm (exceeding the absorption spectrum of InSe), critical BTBT memory shows positive responses arising from the BP channel. Moreover, when *V_g_* = 20 V (extremely above *V_NDR_*), there is no BTBT due to no available states to tunnel to. Besides, the storage of holes is also suppressed under the positive back gate control. Although negative photoresponse persists, the falling time exhibits little difference compared with the rising time. The optoelectronic performances to different wavelengths are summarized in **Fig. S13 d**.


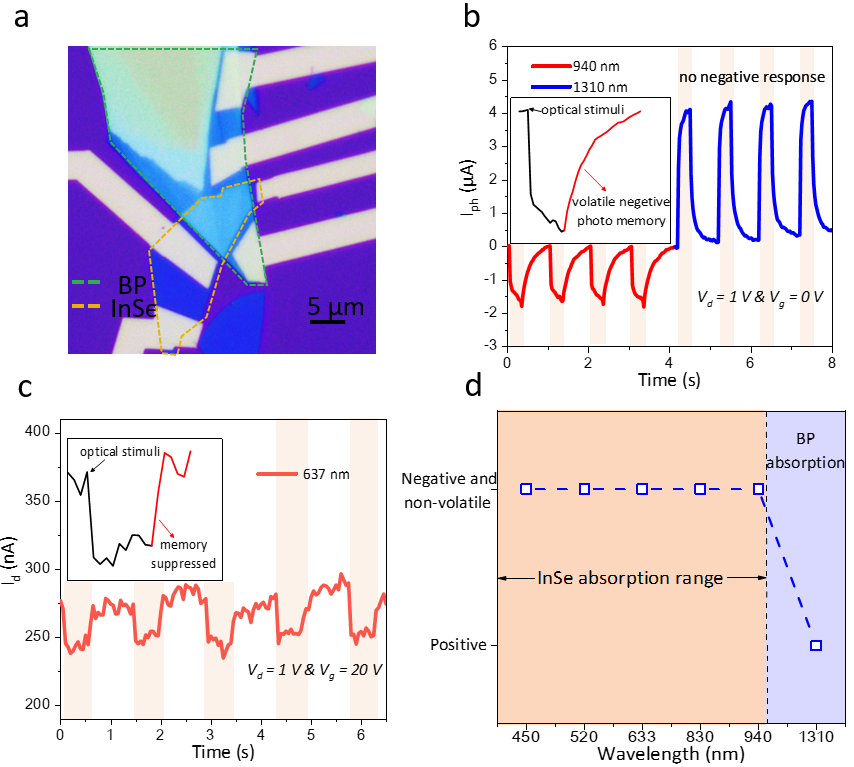


**Fig. S13 | The optoelectronic performance under different wavelengths and gate voltages. a,** The optical paragraph of BP on the InSe device. **b,** Demonstration of the photoresponse of critical BTBT memory to 940 nm (red line) and 1310 nm (blue line) at *V_d_* =1 V, *V_g_*= 0 V. Vertical orange columns represent the light states (940nm and 1310 nm respectively), the duration of laser is 300 ms. Inset: Enlarged view of the photoresponse in one cycle. The black arrow indicates the moment of laser stimulation. The red line is the falling edge. **c,** Demonstration of the photoresponse of critical BTBT memory to 637 nm at *V_d_* = 1 V, *V_g_* = 20 V. Vertical orange columns represent the light states (637 nm); the duration is 500 ms. Inset: Enlarged view of the photoresponse in one cycle. The black arrow indicates the moment of laser stimulation. The red line is the falling edge. **d,** The optoelectronic effect under different incident wavelengths.

**Section 7. Comparing performances of similar structures**

To exclude other potential factors that may also induce photomemory characteristics, we fabricate similar devices with different materials (MoTe_2_ and WSe_2_); the comparison results are shown in **Table 1**. There are two main reasons for utilizing MoTe_2_ and WSe_2_:

1) Both are bipolar materials like BP.

2) Neither can satisfy the *critical BTBT condition* with BP or InSe.

The first reason ensures a significant change when photomemory current exists, and the second reason is to distinguish the BTBT impact on photomemory current. The detailed discussions are as follows.

**Supplementary table 1.** Comparing performances of similar structures but with different materials

**
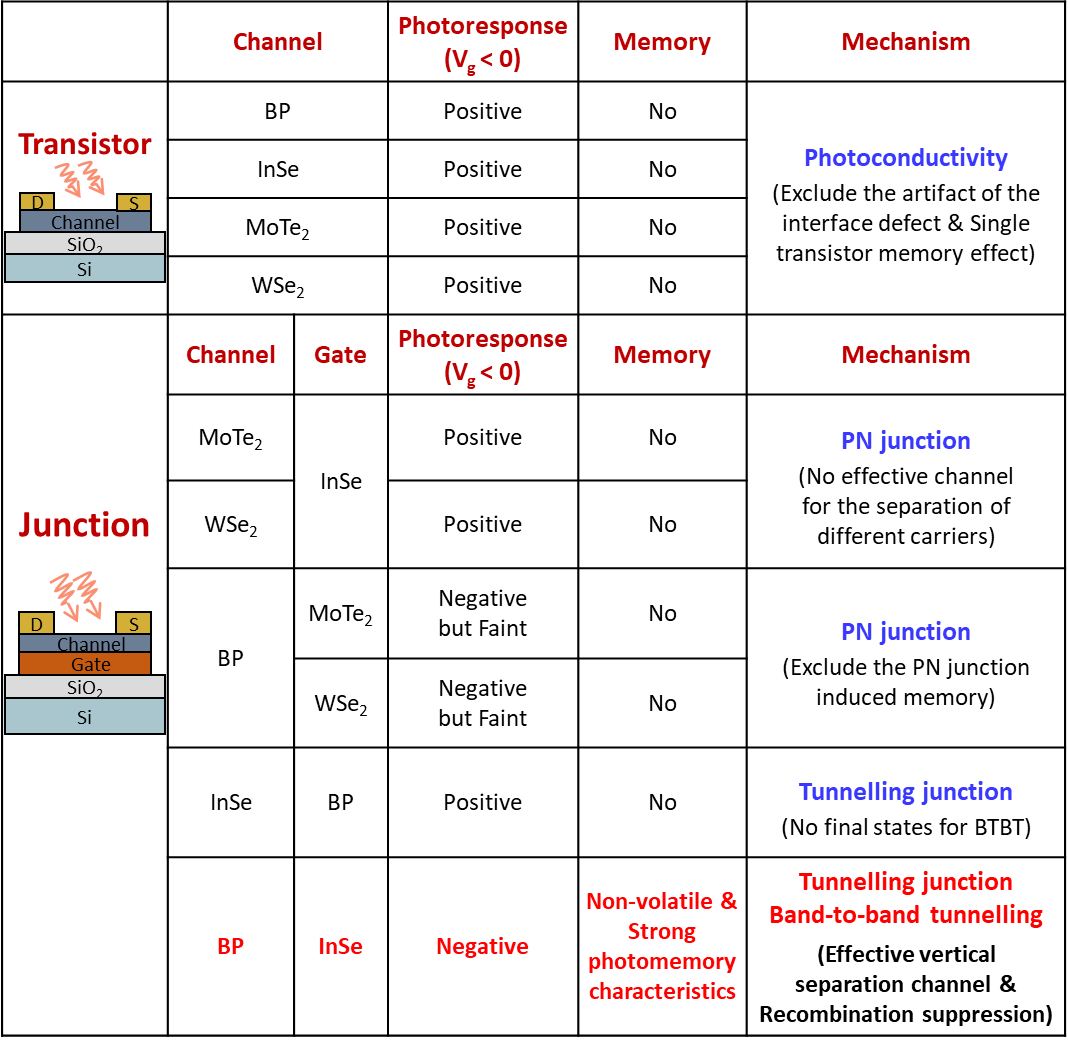
**

In the following discussion on **Supplementary** **Table 1**, orange columns always represent the light states. Meanwhile, the white column represents the dark states (0.5 s). The optical stimuli are 633 nm with a sufficient wide pulse width (0.5 s) and laser power (4 mW). Based on the results in the manuscript, this optical stimulus is strong enough to cause a magnitude change in the critical BTBT memory current.

**Ⅰ. Artefact of the interface defects**

First, to demonstrate the artefact of interface defects, we fabricate the BP and InSe transistors by mechanical exfoliation in the glove box. The optical photograph is shown in **Fig. S14**. The photoresponses of both transistors are positive and stable with no memory characteristics.

Compared with the optoelectronic memory works based on interface defects^8^, if the photomemory current of critical BTBT memory is arising from an artefact of interface defects, the transistors show volatile or nonvolatile noticeable memory characteristics under the laser stimulus. While for BP and InSe transistors, there is no significant change in photocurrent under multiple pulse cycles or obvious multi-states in the current of dark states, which confirmed that the artefact of interface defects is negligible in photomemory characteristics of critical BTBT memory.


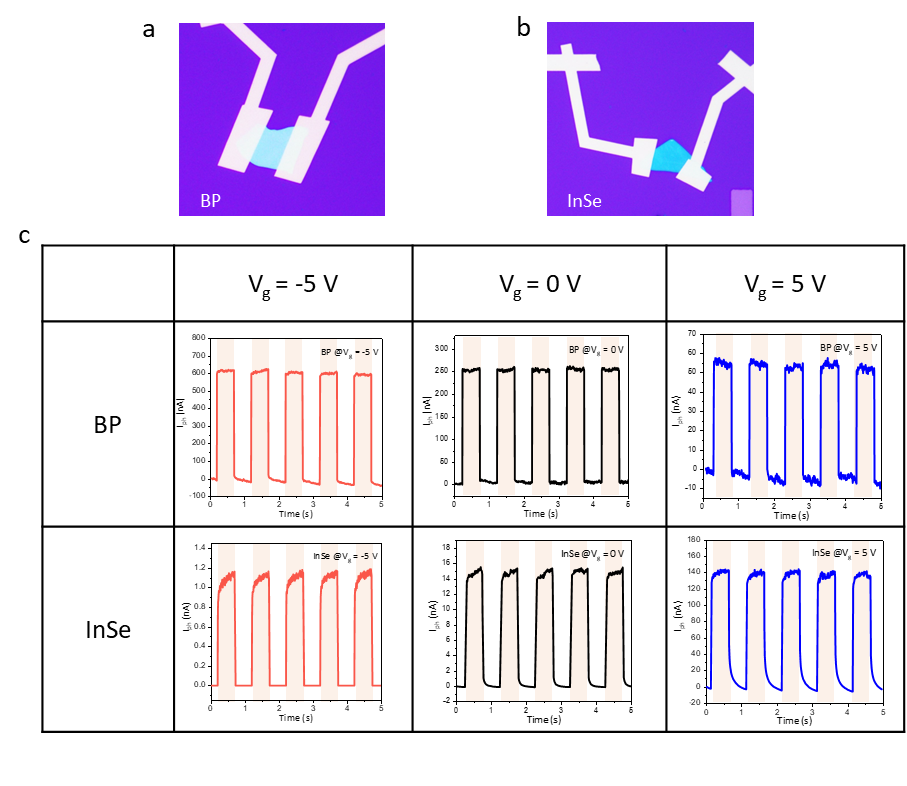


**Fig. S14 | Photoresponse of BP and InSe transistors.** **a,** Optical photograph of the BP transistor. **b,** Optical photograph of the InSe transistor. **c,** Photocurrent of double transistors under different back gate voltages. Regardless of the back gate direction, the positive photoresponse is stable, and there are no significant memory characteristics for either transistor.

**Ⅱ. Photoresponse on MoTe_2_ (or WSe_2_) on InSe device**

Second, we replace BP with MoTe_2_ or WSe_2_ in critical BTBT memory to demonstrate its optical performance (the channel is MoTe_2_ or WSe_2_, and the gate is InSe). In such devices, due to the interface-blocking effect of InSe, the current in the vertical direction is small (reaching the limitation of the experimental instrument). No effective channel for different carriers’ vertical separation accelerates the recombination of different carriers in the gate. Therefore, the photocurrent all arises from the channel and shows no memory characteristics.


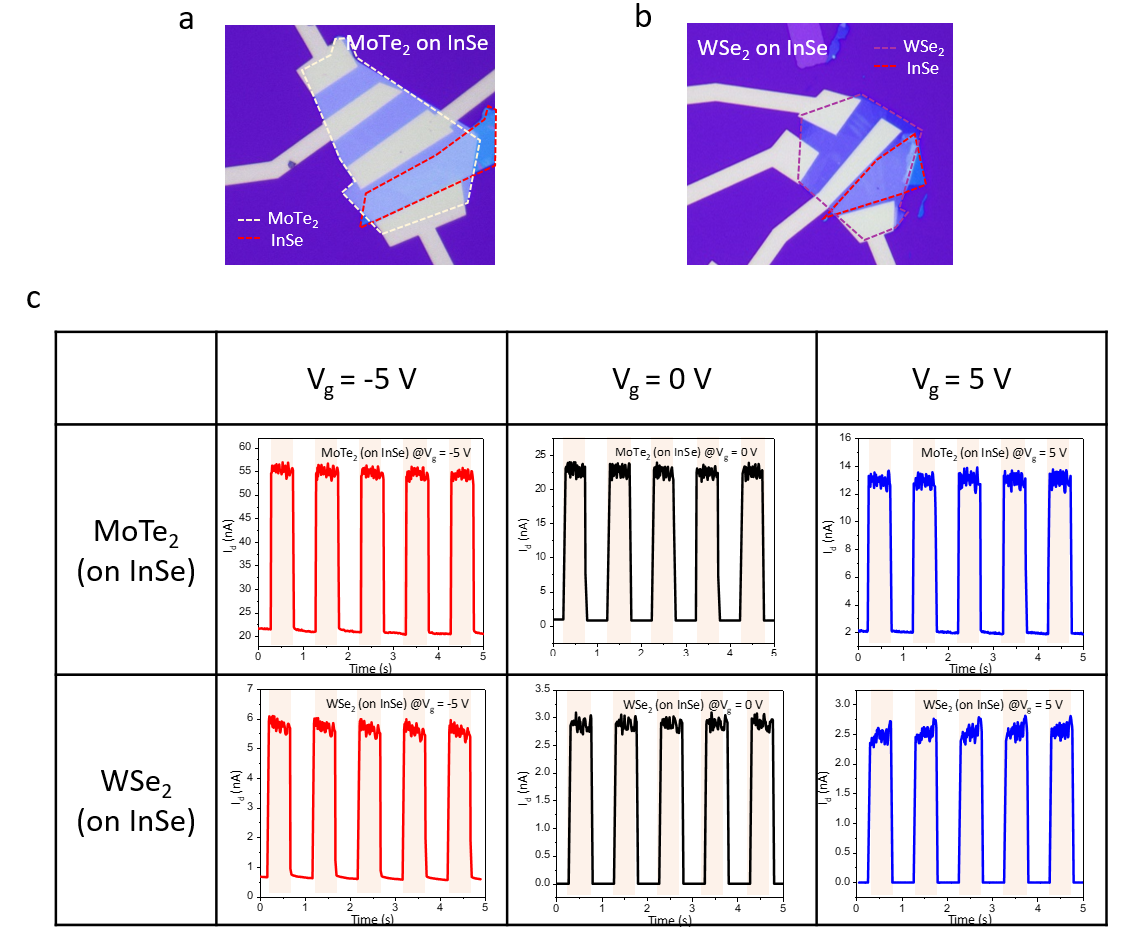


**Fig. S15 | Photoresponse of MoTe_2_ (or WSe_2_) on InSe device.** **a,** Optical photograph of the MoTe_2_ on InSe device. **b,** Optical photograph of WSe_2_ on InSe device. **c,** The table represents the photocurrent of double devices under different back gate voltages. Regardless of the back gate direction, the positive photoresponse is stable with no significant memory characteristics for double devices.

**Ⅲ. Photoresponse on BP on MoTe_2_ (or WSe_2_) device**

Third, we replace InSe with MoTe_2_ or WSe_2_ in critical BTBT memory to demonstrate its optical performance (the channel is BP, and the gate is MoTe_2_ or WSe_2_). The vertical separation channel is established through the PN junction. Although sufficient light stimuli are applied to the junction, there is merely a faint photoresponse, as shown in **Fig. S16**. Note that the BP transistor only shows a positive photoresponse; the negative response must be from the PN junction-induced effect. Photogenerated holes are accumulated under the negative back gate voltage, decreasing the p-type channel current.

However, the PN junction demonstrates a significantly higher dark current, surpassing critical BTBT memory by more than 4 orders of magnitude^9^. This elevated dark current facilitates extensive recombination, ultimately resulting in a faint photoresponse with no memory characteristics. In contrast, for critical BTBT memory, on the one hand, there are few holes supposed to tunnel through the barrier in the absence of stimulus; on the other hand, the spatial recombination is suppressed by delicate band alignment, both of which ensure a nonvolatile and strong photomemory characteristics.

1. **BP on MoTe_2_ device:**


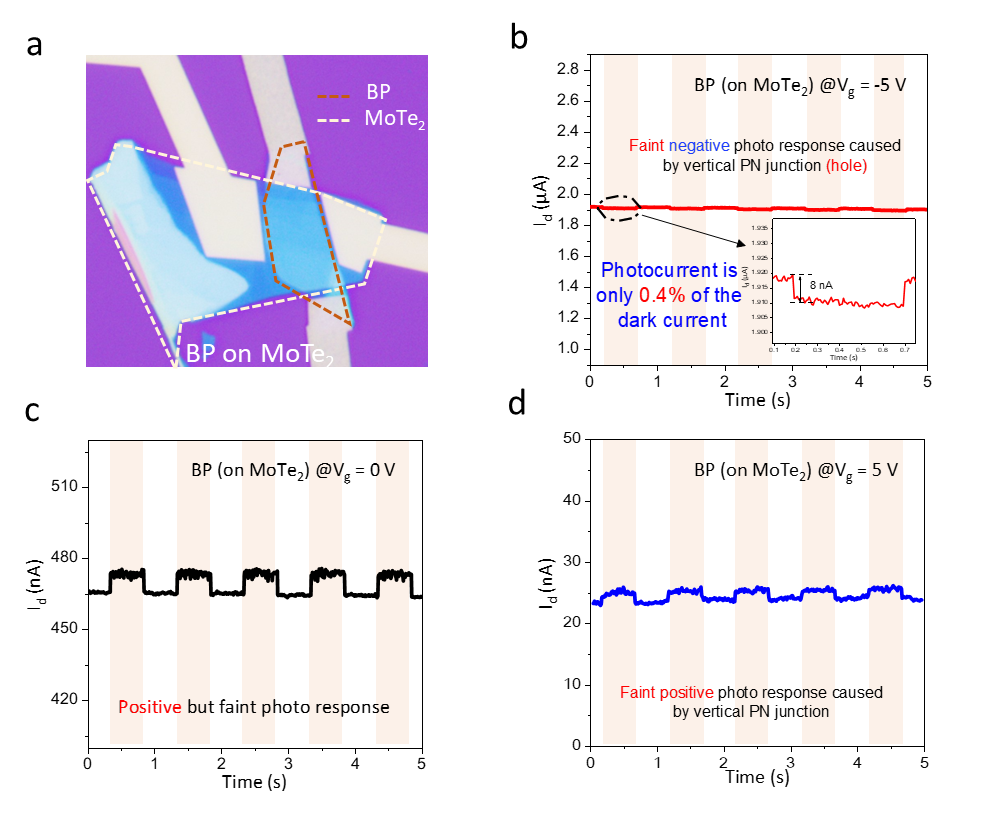


**Fig. S16 | Photoresponse of MoTe_2_ on InSe device.** **a,** Optical photograph of BP on MoTe_2_ device. **b,** A faint negative photoresponse caused by the vertical PN junction under *V_g_* = -5 V, meaning the holes are accumulated in the gate. **c,** No obvious photomemory characteristics under *V_g_* = 0 V. **d,** A faint positive photoresponse under *V_g_* = 5 V.

1. **BP on WSe_2_ device**:


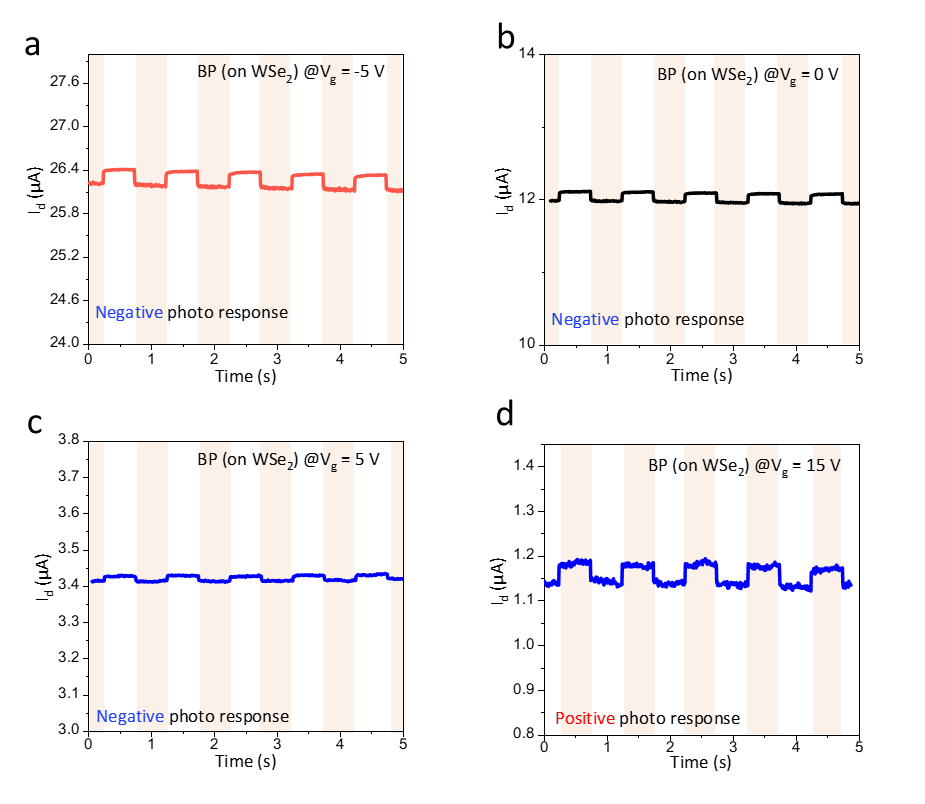


**Fig. S17 | Photoresponse of WSe_2_ on InSe device.** **a,** Negative photoresponse caused by the vertical PN junction under *V_g_* = -5 V. **b,** Negative photoresponse caused by the vertical PN junction under *V_g_* = 0 V. **c,** A faint negative photoresponse caused by the vertical PN junction under *V_g_* = 5 V, meaning the holes are still accumulated in the gate. **d,** Positive photoresponse caused by the vertical PN junction under *V_g_* = 15 V, meaning the electrons are accumulated in the gate.

**Ⅳ. Photoresponse of InSe on BP device**

Then, as for the tunnelling junction-based devices, there are also double situations. For InSe on the BP device, the *critical BTBT condition* is also satisfied. However, BTBT only occurs in this scenario with the positive back gate voltage to tunnel to the available final state. The holes accumulate on the surface of InSe and are collected by the electrode, leading to a positive photocurrent with no memory characteristic.


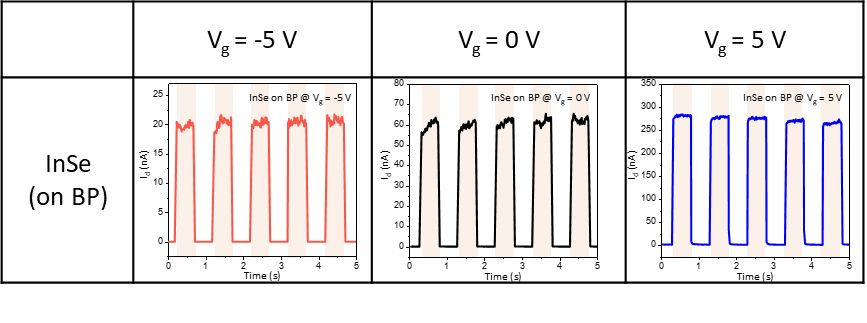


**Fig. S18 | Photoresponse of InSe on BP device.** Regardless of the back gate direction, the positive photoresponse of InSe on the BP device is stable with no significant memory characteristics.

**Section 8. Positive photomemory current of *critical BTBT-based* semi-floating gate device**

Inspired by the design of Si-based semi-floating gate devices^10^, we fabricate the *critical band-to-band tunnelling-based* semi-floating gate device to obtain a positive photomemory current. Fig. S19 shows that critical BTBT memory works as a semi-floating gate at the top of the structure. Through the optical writing, the remaining photogenerated holes in InSe increase the current of the n-type MoS_2_ channel. Critical band-to-band tunnelling-based semi-floating gate device has cumulative positive photoconductivity with multilevel states under 100 μs laser stimuli. The erasing pulse width is constrained to 100 ms by the B1500 and the parasitic capacitance of the Probe Station.


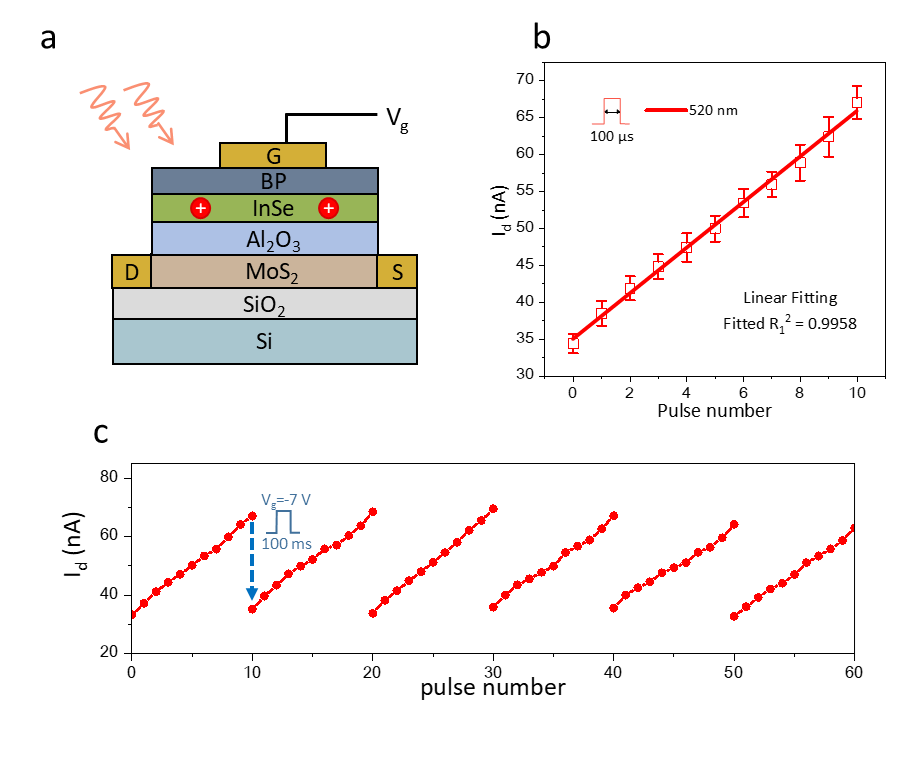


**Fig. S19 | Reconfigurable positive photomemory characteristics of *critical BTBT-based* semi-floating gate device.** Critical tunnelling-based semi-floating gate device works at *V_d_* = 0.8 V, *V_g_* = 3 V. All the output current is read at approximately 0.5 s after each laser stimulus. **a,** *Critical BTBT-based* semi-floating gate device structure. **b,** Statistical distribution of the output current under the varying amounts of laser pulse stimuli in ten times experiments. The laser pulse is 520 nm with 100 μs duration and 1 s interval. The erasing electrical pulse is *V_g_* = -7 V for 100 ms. The solid line shows the linear variation, the linear fitting *y = b+a×x*, where *b* is 34.98, *a* is 30.89, and *R^2^*=0.9958. **c,** Positive photomemory current of *critical BTBT-based* semi-floating gate device in several cycles.

**Section 9. Laser pulse characterization**

The sub-microsecond laser pulse is first generated by Rigol DG 5071 in the form of a digital electrical signal. Then it controls the OBIS laser to generate a 520 nm laser pulse. The laser performance is calibrated by a commercial Si-based photodetector and the output signal is captured by a Tektronix MDO34 oscilloscope.


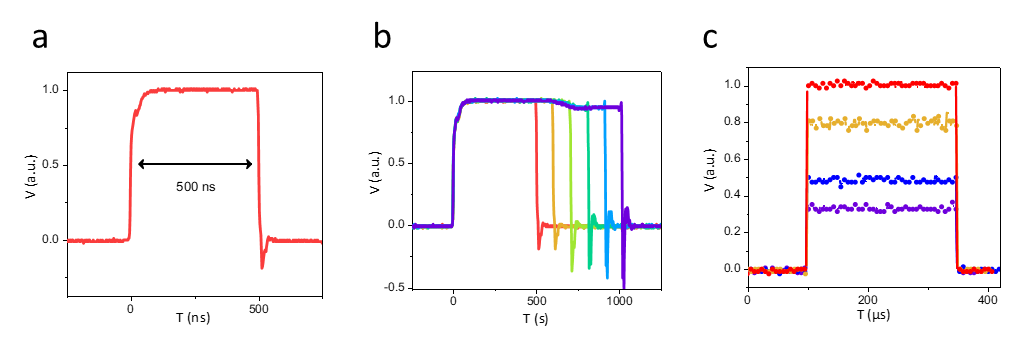


**Fig. S20 |** Demonstration of the sub-microsecond laser pulse width and pulse power. **a,** The demonstration of 500 ns laser pulse width. **b,** Laser pulse power at 500 ns - 1000 ns with no significant change. **c,** Variation of optical power using attenuators.

**Section 10. Negative photomemory characteristics of critical BTBT memory**

**Ⅰ. Visible negative photomemory characteristics**

**Fig. S21** shows the accurate photomemory current value of critical BTBT memory in **Fig. 2a**. We use $R= I_{pmc}/(P\times\frac{A_{device}}{A_{laser}})$ to demonstrate the responsivity of critical BTBT memory, where *R* represents the photoresponsivity, *I_pmc_* represents the photomemory current, *P* represents the laser power, *A_device_* and *A_laser_* refer to the device area and laser spot area, respectively. In this work, the power of the laser is 960 μW, and the critical BTBT memory area is 5 μm × 10 μm. For critical BTBT memory, the output current *I_pmc_* do not directly stem from the photoelectric conversion process but rather from the manipulation of stored holes. The gain is achieved through gate manipulation, resulting in increased responsivity. In such a case, the responsivity surpasses the limits of quantum efficiency^11^.


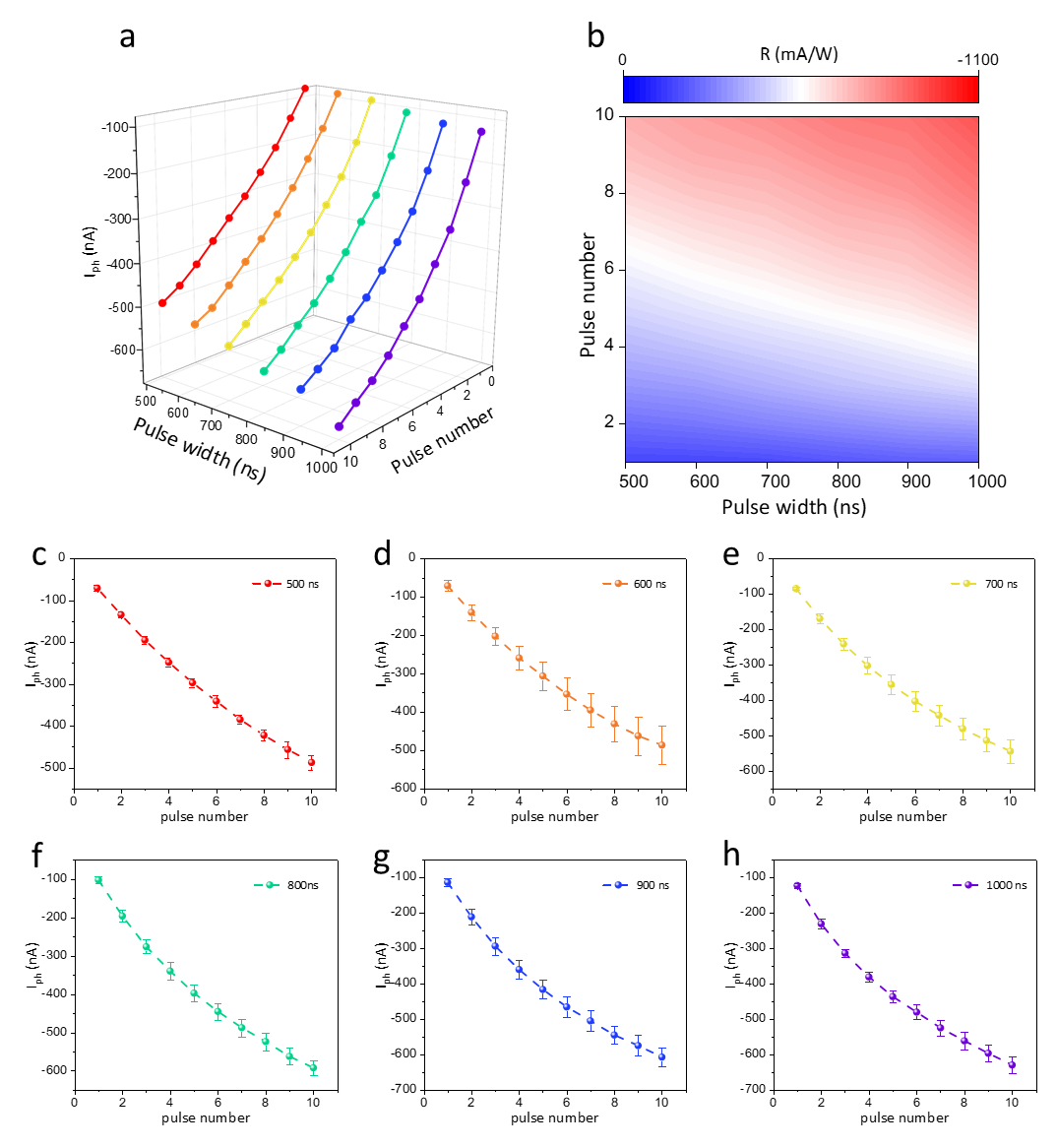


**Fig. S21 | Negative photomemory characteristics of critical BTBT memory to the visible laser pulse.** Critical BTBT memory works at *V_d_* = 1 V, *V_g_* = -5 V. All the output current is read at approximately 0.1 s after each laser stimulus. **a,** Negative photomemory current of critical BTBT memory to 520 nm laser pulse. The interval of the laser pulse is 0.2 s. **b,** The responsivity of critical BTBT memory. **c-h,** Statistical distribution of the negative photomemory current to laser pulse at 500-1000ns.

**Ⅱ. NIR negative photomemory characteristics**

**Fig. S22 | Negative photomemory characteristics of critical BTBT memory to the NIR laser pulse.** The photomemory properties are measured at *V_d_* = 1 V, *V_g_* = -5 V. The blue line and red line represent the photomemory characteristics of the 830 nm and 940 nm laser pulse, respectively. Each laser pulse lasts 100 μs with a 1 s interval.

**Section 11. Stability of critical BTBT memory**

**Ⅰ. Time stability of critical BTBT memory**

Given the fact that certain protective layers, such as Al_2_O_3_ or HfO_2_^12,13^, may exhibit a doping effect on 2D materials, we simplified the device structure with no protective encapsulation. In contrast, the operating environment is taken seriously to eliminate the severe degradation of the BP channel, as illustrated in the method. With a high vacuum experiment environment and nitrogen preservation, critical BTBT memory is protected.

**
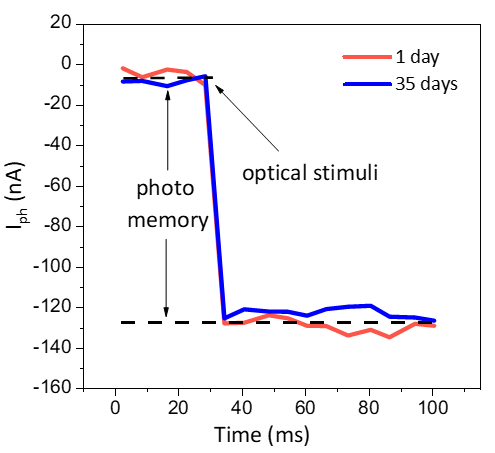
**

**Fig. S23 | Comparison of the photomemory current after 1 day and 35 days of device fabrication to a 940 nm stimulus.** There is no significant difference in the comparison after 1 day and after 35 days, indicating the time stability of critical BTBT memory.

**Ⅱ. Critical BTBT memory with protective encapsulation**

Under the optical stimuli, critical BTBT memory with hBN as the protective encapsulation also exhibits photomemory characteristics to 633 nm optical stimulus.


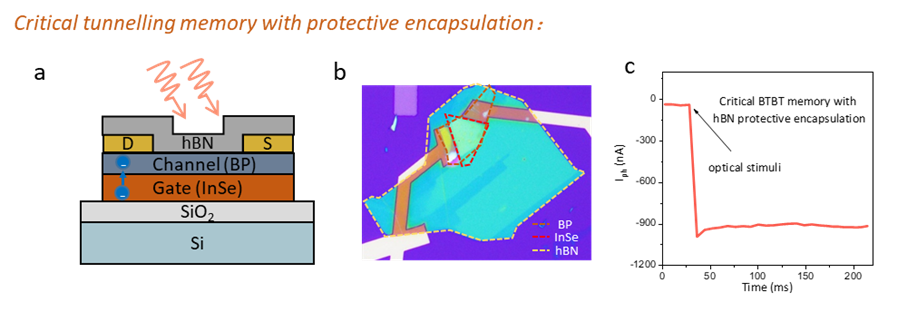


**Fig. S24 | Critical BTBT memory with protective encapsulation.** a, The device structure of critical BTBT memory with hBN encapsulation. b, Corresponding optical photograph. c, Negative photomemory characteristics of critical BTBT memory with protective encapsulation, the photomemory current are measured at *V_d_* = 1 V, *V_g_* = -5 V. All the fabrication process is in the glove box.

**Section 12. The endurance of critical BTBT memory**

**Fig. S26** demonstrates the endurance of critical BTBT memory for 50 cycles of the relatively long optical writing or electrical erasing pulse.


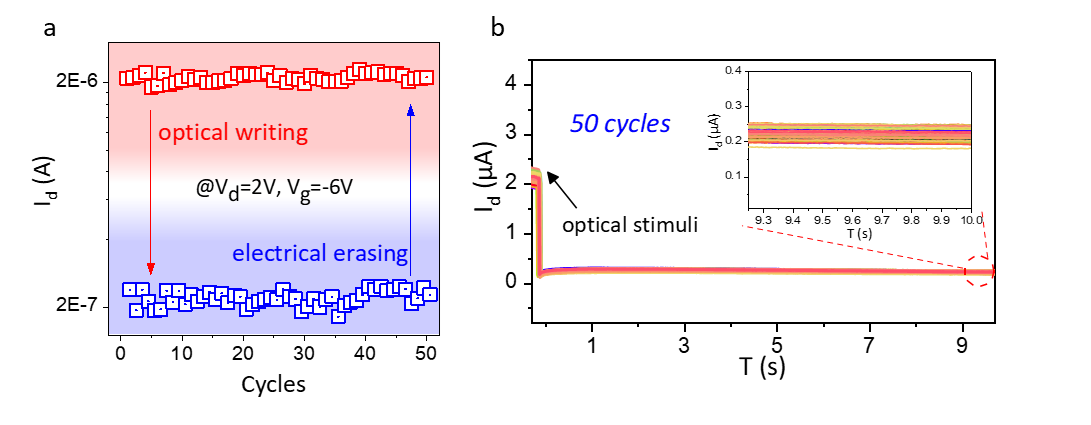


**Fig. S25 | The endurance of critical BTBT memory for 50 cycles of writing/erasing pulse.** The output current of critical BTBT memory is obtained at *V_d_* = 2 V, *V_g_* = -6 V. **a,** The laser stimulus is 520 nm with a 2 ms duration. The erasing electrical pulse is *V_g_* = 10 V for 100 ms. Red and blue points represent the output current of critical BTBT memory at the initial and the tenth second after laser stimuli, respectively. **b,** The corresponding current in the time domain.

**Section 13. Analytic model of InSe/BP heterojunction energy band**

The specific energy barrier distribution and the slope of the energy band for small barriers directly affect various current components in the InSe/BP heterojunction, such as the tunnelling current being strongly influenced by the initial positions and the corresponding position of the final states. Thus, the relative positions and slopes of the conduction and valence bands of heterojunctions need to be modelled accurately. With the condition of small injection, the Debye length for InSe and BP are much larger than the thickness of respective layers. Thus, a linear function is a good approximation to model both materials' conduction and valance bands. Due to the poor conduction between BP and InSe, boundary conditions between all three interfaces along the normal direction are

$\left(\mathbf{D}_{bP}-\mathbf{D}_{InSe}\right)\cdot\mathbf{n}=0$,

$\left(\mathbf{D}_{InSe}-\mathbf{D}_{SiO_{2}}\right)\cdot\mathbf{n}=0$,

where $\mathbf{D}_{bP}$, and are the electric displacement vector of BP, InSe, and SiO_2_, respectively. $\mathbf{n}$ is the normal vector perpendicular to the interface. The potential drop is approximately zero in heavily doped silicon substrates. The boundary condition between SiO_2_ and Si is neglected. Taking the energy bands, which are both linear functions, the electric displacement vector is constant in InSe and BP. The relationship between the electric field and the applied gate voltage $V_{Gate}$ can be written as

$V_{Gate}=\frac{L_{bP}}{\varepsilon_{bP}}\left(\mathbf{D}_{bP}\cdot\mathbf{n}\right)+\frac{L_{InSe}}{\varepsilon_{InSe}}\left(\mathbf{D}_{InSe}\cdot\mathbf{n}\right)+\frac{L_{SiO_{2}}}{\varepsilon_{SiO_{2}}}\left(\mathbf{D}_{SiO_{2}}\cdot\mathbf{n}\right)$,

where $L_{bP}$, $L_{InSe}$, and $L_{SiO_{2}}$ are the thickness of BP, InSe, and SiO_2_, respectively. Solving the equations above, we have

$\tilde{k}_{bP}=\frac{\mathbf{E}_{bP}\cdot\mathbf{n}}{V_{Gate}}L_{bP}=\frac{L_{bP}}{\varepsilon_{bP}}\frac{1}{\frac{L_{bP}}{\varepsilon_{bP}}+\frac{L_{InSe}}{\varepsilon_{InSe}}+\frac{L_{SiO_{2}}}{\varepsilon_{SiO_{2}}}}$,

$\tilde{k}_{InSe}=\frac{\mathbf{E}_{InSe}\cdot\mathbf{n}}{V_{Gate}}L_{InSe}=\frac{L_{InSe}}{\varepsilon_{InSe}}\frac{1}{\frac{L_{bP}}{\varepsilon_{bP}}+\frac{L_{InSe}}{\varepsilon_{InSe}}+\frac{L_{SiO_{2}}}{\varepsilon_{SiO_{2}}}}$,

$\tilde{k}_{SiO_{2}}=\frac{\mathbf{E}_{SiO_{2}}\cdot\mathbf{n}}{V_{Gate}}L_{SiO_{2}}=\frac{L_{SiO_{2}}}{\varepsilon_{SiO_{2}}}\frac{1}{\frac{L_{bP}}{\varepsilon_{bP}}+\frac{L_{InSe}}{\varepsilon_{InSe}}+\frac{L_{SiO_{2}}}{\varepsilon_{SiO_{2}}}}$,

where $\mathbf{E}_{bP}$, $\mathbf{E}_{InSe}$, and $\mathbf{E}_{SiO_{2}}$ are the electric field vector of BP, InSe, and SiO_2_, respectively. $\tilde{k}_{bP}$, $\tilde{k}_{InSe}$, and $\tilde{k}_{SiO_{2}}$ are the dimensionless slope of the energy band in BP, InSe, and SiO_2_, respectively. Substituting material properties of BP, InSe, and SiO_2_, we have $\tilde{k}_{bP}=1.221\times10^{-2}$ and $\tilde{k}_{InSe}=6.002\times10^{-2}$, which coincides with the semiconductor simulation result. Assuming the interface between InSe and BP is at the *yOz* plane, i.e., at *x* = 0, the conduction band $E_{c,bP}\left(x,V_{Gate}\right)$ and valance band $E_{v,bP}\left(x,V_{Gate}\right)$ of BP could be written as

$E_{c,bP}\left(x,V_{Gate}\right)=E_{v,bP}\left(0\right)+E_{g,bP}-k_{bP}\left(V_{Gate}\right)x$,

$E_{v,bP}\left(x,V_{Gate}\right)=E_{v,bP}\left(0\right)-k_{bP}\left(V_{Gate}\right)x$.

where

$$k_{bP}\left(V_{Gate}\right)=\frac{\tilde{k}_{bP}}{L_{bP}}\left(V_{Gate}-V_{th}\right)$$

is the gate-related slope of the energy band of BP. $V_{th}=2.16 \text{V}$ is the flat band value of the gate voltage, derived from the simulation result of saturation behavior of the number density of stored holes. Similarly, the conduction band $E_{c,InSe}\left(x,V_{Gate}\right)$ and valance band $E_{v,InSe}\left(x,V_{Gate}\right)$ of InSe could be written as

$E_{c,InSe}\left(x,V_{Gate}\right)=E_{c,InSe}\left(0\right)-k_{InSe}\left(V_{Gate}\right)x$,

$E_{v,InSe}\left(x,V_{Gate}\right)=E_{c,InSe}\left(0\right)-E_{g,InSe}-k_{bP}\left(V_{Gate}\right)x$,

where

$k_{InSe}\left(V_{Gate}\right)=\frac{\tilde{k}_{InSe}}{L_{InSe}}\left(V_{Gate}-V_{th}\right)$.

Introducing $E_{d}\equiv E_{v,bP}\left(0\right)-E_{c,InSe}\left(0\right)$ and assuming $E_{c,InSe}\left(0\right)=0$, we have

$E_{c,bP}\left(x,V_{Gate}\right)=E_{d}+E_{g,bP}-k_{bP}\left(V_{Gate}\right)x$,

$E_{v,bP}\left(x,V_{Gate}\right)=E_{d}-k_{bP}\left(V_{Gate}\right)x$,

and

$E_{c,InSe}\left(x,V_{Gate}\right)=-k_{InSe}\left(V_{Gate}\right)x$,

$E_{v,InSe}\left(x,V_{Gate}\right)=-E_{g,InSe}-k_{bP}\left(V_{Gate}\right)x$.

**Section 14. Hole diffusion current of InSe/BP heterojunction**

At different positions of InSe, the density of holes in the valance band is

$p_{InSe}\left(x,V_{Gate},E_{Fp,InSe}\right)=N_{v,InSe}\exp\left(-\frac{E_{Fp,InSe}-E_{v,InSe}\left(x,V_{Gate}\right)}{k_{B}T}\right)$.

where $N_{v,InSe}$ is the density of state of the valance band of InSe. Substituting the analytic model of the energy band of InSe, we have

$$p_{InSe}\left(x,V_{Gate},E_{Fp,InSe}\right)=N_{v,InSe}\exp\left(\frac{E_{v,InSe}\left(0\right)}{k_{B}T}\right)\exp\left(-\frac{E_{Fp,InSe}}{k_{B}T}\right)\exp\left(-\frac{k_{0}x\left(V_{Gate}-V_{0}\right)}{k_{B}Tx_{L}}\right)$$

Integrating the hole density $p_{InSe}\left(x,V_{Gate},E_{Fp,InSe}\right)$ along the thickness direction, the surface density of the hole $Q_{p}\left(V_{Gate},E_{Fp,InSe}\right)$ can be expressed and explicitly calculated

$$\begin{matrix} Q_{p}\left(V_{Gate},E_{Fp}\right)= \int_{0}^{x_{L}} p_{InSe}\left(x,V_{Gate},E_{Fp,InSe}\right)dx \\ \\ = \frac{N_{v,InSe}k_{B}Tx_{L}}{k_{0}\left(V_{Gate}-V_{0}\right)}\exp\left(\frac{E_{v,InSe}\left(0\right)}{k_{B}T}\right)\exp\left(-\frac{E_{Fp,InSe}}{k_{B}T}\right)\left[1-\exp\left(-\frac{k_{0}x\left(V_{Gate}-V_{0}\right)}{k_{B}Tx_{L}}\right)\right] \end{matrix}$$

The hole diffusion current at the BP/InSe interface is affected by the contact conditions. Phenomenologically, this could be described by a low mobility at the interface $\tilde{\mu}_{p}$ compared with a much higher mobility in the interior of InSe. Thus, the hole diffusion current at the BP/InSe interface

$$J_{p}^{diff}=\frac{k_{B}T}{e}\tilde{\mu}_{p}\frac{dp}{dx}\approx\frac{k_{B}T}{e}\tilde{\mu}_{p}\frac{p_{InSe}\left(0+\delta x\right)-p_{bP}\left(0-\delta x\right)}{2\delta x}$$

where $\delta x$ is a small quantity. In the above equation, the derivative is rewritten as a difference for that quasi-equilibrium state, which is formed separately in both InSe and BP. To obtain the expression of current $J_{p}^{diff}$ about $Q_{p}$ and $V_{Gate}$, the $Q_{p}\left(V_{Gate},E_{Fp,InSe}\right)$ is numerically solved, and the expression of $E_{Fp,InSe}$ about $Q_{p}$ and $V_{Gate}$ is then obtained based on the high-precision linear interpolation and further substituted into the simplified current proportional relationship.

**Section 15. Band-to-band tunnelling between InSe and BP**

Since the diffusion and drift currents are suppressed in the heterojunction of InSe/BP, the tunnelling current becomes the dominant charge component across the heterojunction interface. From the analytic model of the InSe/BP heterojunction energy band, as the energy band changes from a negative gate voltage to a positive bias voltage, the slope of the energy band of both InSe and BP changes from positive to negative and three typical states occur.


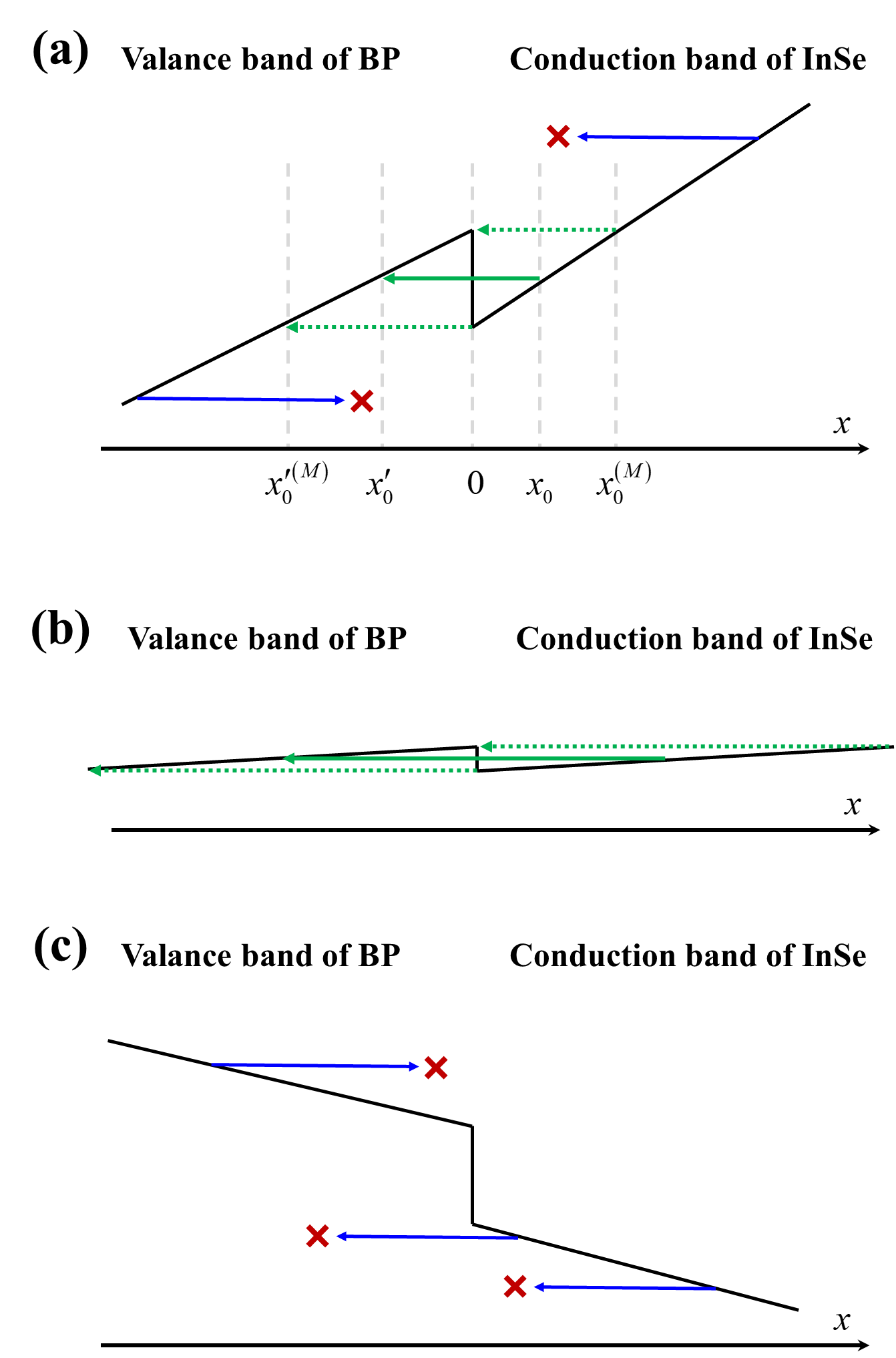


**Fig. S26 | Band-to-band tunnelling under negative gate voltage. a,** In the case of large negative gate voltage, both BP and InSe energy bands are severely tilted, and only small regions in BP and InSe can tunnelling. **b,** In the case of the maximum tunnelling current, the energy range of the conduction band of InSe coincides with the valence band of BP. **c,** In the case of large positive gate voltage, the tunnelling is cut off.

Since the tunnelling process satisfies energy conservation, the electrons at a point $x_{0}$in the conduction band of InSe could only tunnel to the position $x'_{0}$ in the valance band of BP. Thus, the following relation is satisfied

$E_{c,InSe}\left(x_{0},V_{Gate}\right)=-k_{InSe}\left(V_{Gate}\right)x_{0}=E_{v,bP}\left(x',V_{Gate}\right)=E_{d}-k_{bP}\left(V_{Gate}\right)x'_{0}$.

Solve this equation, and we have

$\frac{k_{InSe}\left(V_{Gate}\right)}{k_{bP}\left(V_{Gate}\right)}x_{0}+\frac{1}{k_{bP}\left(V_{Gate}\right)}E_{d}=x'_{0}$.

Based on quantum mechanics, under the assumption that the electron wave function has the form of a plane wave, the tunnelling rate of electron tunnelling through the potential barrier from $x$ to $x'$ can be written by an integral

.

In the heterojunction of InSe/BP, we have

$\sqrt{U\left(x\right)-E}=\sqrt{E_{d}+k_{InSe}\left(V_{Gate}\right)x_{0}-k_{bP}\left(V_{Gate}\right)x}$.

The electron tunnelling from the conduction band of InSe to the position $x'_{0}$ in the valance band of BP could be written as

.

Then, the integral could be calculated explicitly,

.

In the conduction band of InSe, the largest $x_{0}$ could tunnel to the valance band of BP, satisfying the following relationship

$x_{0}<-\frac{1}{k_{InSe}\left(V_{Gate}\right)}E_{d}=x_{0}^{\left(M\right)}$,

which gives $x_{0}$ an upper bound. The value of the tunnelling current is proportional to the density of electrons at $x_{0}$ and the density of holes at $x'_{0}$, so the tunnelling current can be written in the form

.

In the above expressions, it is assumed that the thicknesses of both InSe and BP are infinite. This assumption does not affect the analysis of the BTBT current behaviour in the hysteresis curve. This assumption can only affect a small region of gate voltage. This region starts from the right end of the conduction band of InSe, having equal energy to the right end of the valence band of BP, and ends with the right end of the conduction band of InSe, having equal energy to the left end of InSe. The width of this region is $E_{d}/\tilde{k}_{InSe}\approx0.017 \text{V}$ is much larger than the voltage step 0.2 V. When the gate voltage is negative, the tunnelling current from the BP side to the InSe side is much smaller than that from InSe to BP. In the time-domain simulation of the energy band and the saturation behavior of hole storage with negative gate voltage, the tunnelling current from the BP side to the InSe side is neglected. Noticing that band-to-band tunnelling mainly occurs near the interface at high negative gate voltage, the number density of holes in both BP and InSe sides are replaced with equivalent values. Thus, we have

.

The band-to-band tunnel recombination in the time-domain simulation of the energy band and the saturation behavior simulation is based on this expression, in which $\tilde{A}=1\times10^{-10}$ is chosen. The function $F\left(V_{Gate}\right)$ is defined as

.

Substituting the explicit expression of $x_{0}^{\left(M\right)}$ into the integral we have

,

where

$$E_{n}=\int_{1}^{\infty} \frac{e^{-zt}}{t^{n}}dt$$

is the exponential integral function. It should be noticed that the exponential integral function has a branch cut discontinuity at the nonpositive part of the real axis in the complex plane. Thus, only the positive value of the variable is acceptable.

Noticing $E_{v,bP}\left(0,V_{Gate}\right)-E_{c,InSe}\left(0,V_{Gate}\right)=E_{d}$, we have

$I^{\left(-\right)}\propto\exp\left(\frac{E_{fe,InSe}-E_{fh,bP}}{k_{B}T}\right)F\left(V_{Gate}\right)$,

where the superscript shows the direction of tunnelling. Similarly, we have

$I^{\left(+\right)}\propto-\exp\left(-\frac{E_{fe,InSe}-E_{fh,bP}}{k_{B}T}\right)F\left(V_{Gate}\right)$.

Thus, we could further derive the final form of the total BTBT current

$I_{bbt}=I^{\left(+\right)}+I^{\left(-\right)}\propto\sinh\left(\frac{E_{fe,InSe}-E_{fh,bP}}{k_{B}T}\right)F\left(V_{Gate}\right)$.

As is in the section for diffusion current of holes, to obtain the expression of current $I_{bbt}$ about $Q_{n}$ and $V_{Gate}$, the $Q_{n}\left(V_{Gate},E_{Fn,InSe}\right)$ is numerically solved, and the expression of $E_{Fn,InSe}$ about $Q_{n}$ and $V_{Gate}$ is then obtained based on the high-precision linear interpolation and further substituted into the simplified current proportional relationship.

**Section 16. Optical characterization of critical BTBT memory**

The optical characteristics of optoelectronic memories, such as absorption and quantum efficiency, are critical as they are the decisive factors in the device performance, such as energy consumption, efficiency, and application scope of the device.

**Ⅰ. Absorption characterization of critical BTBT memory**

The critical BTBT memory is transferred on transparent fused silica and encapsulated by the hBN layer for atmospheric absorption measurement as shown in **Fig. S27**. Differential detection methods are used to measure the absolute absorption of each layer, where the transparent fused silica is the standard sample. First, we measure the absorption of the hBN layer as shown in **Fig. S27 b**. Note that the absorption of hBN is always lower than ±0.2%, which could be due to fluctuations in the instrument's measurements. Combined with the transmission and reflection spectra, we obtained the absorbance of the material by using the Beer-Lambert law,$A= -{log}_{10}\left( \frac{T}{1-R} \right)$. Considering the thin thickness and uniformity of the demonstrated device, the normalized absorbance of the InSe layer is calculated by $A_{normalized}= A/T_{thickness}$, shown in **Fig. S27 c**. The normalized absorbance of the InSe layer is on the same order of magnitude compared with the reported work^14^ (at 500 nm to 1000 nm). Note that the InSe layer is the absorber layer in the demonstrated device, the absolute absorption percentage of the InSe layer is shown in **Fig. S27 d**. Although the InSe layer exhibits nearly 10% absorption in the visible range, the absorption decreases sharply in the infrared range. Both its band gap and thickness make it a weak light absorber, which is a common weakness to many 2D materials.


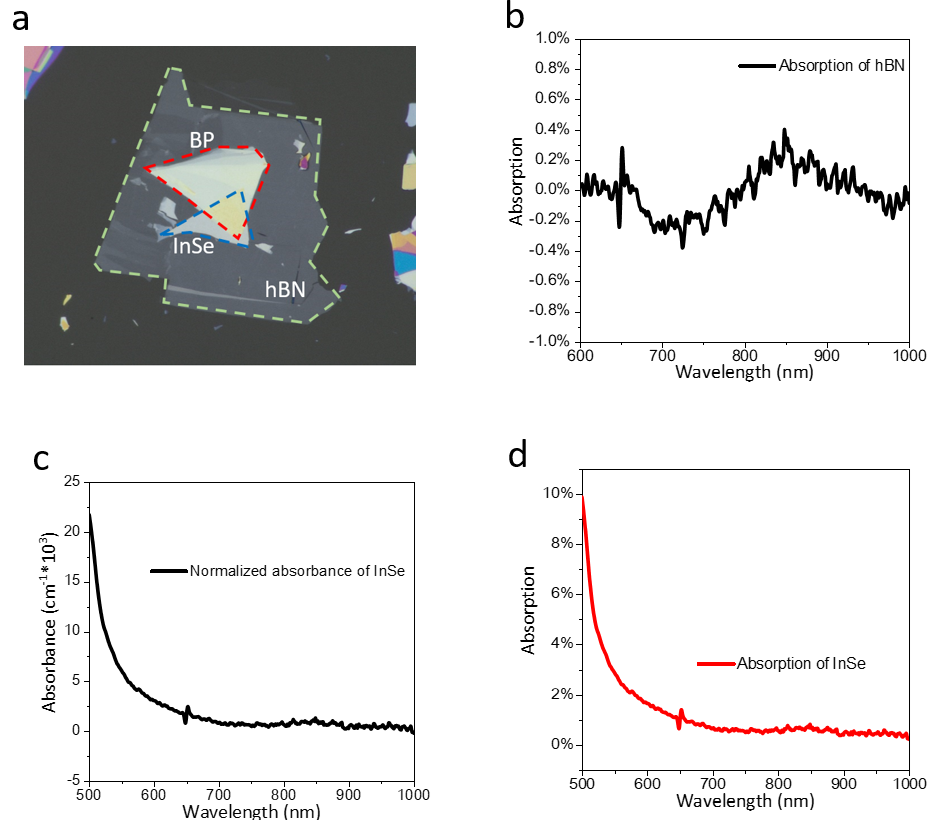


**Fig. S27 | Absorption characterization of the critical BTBT memory. a,** The optical paragraph of critical BTBT memory, which is transferred onto transparent fused silica and encapsulated by the hBN layer for the measurement in atmospheric environments. **b, T**he absorption of the hBN layer. **c,** The normalized absorbance of the InSe layer. **d,** The absolute value of the absorption in the InSe.

**Ⅱ. Quantum efficiency calculation of critical BTBT memory**

Quantum efficiency is another decisive factor for energy consumption. As the absorption is discussed above, here, we calculate the conversion of generated holes and stored holes. The calculation can be divided into three steps:

1) Calculate the number of photons absorbed by the critical BTBT memory in the exposure time and consider that all photons excite electron-hole pairs:

$$n_{generated}=n_{photon}=T_{exposure}*P*\frac{A_{device}}{A_{laser}}*n_{absorption}/hv$$

2) Considering in the demonstrated device, the photo-generated holes stored in the triangular potential well formed by InSe and SiO_2_, controlling the BP channel current, which is similar to parallel plate capacitors, thus the number of stored holes can be calculated by:

$$n_{stored}=C*V$$

*C* represents the capacitance and *V* represents the voltage potential difference. For a cumulative hole-storing process, the physical properties will change over time, which is calculated by COMSOL temporal evolution. Finally, the photo-induced stored holes during an optical stimulus can be calculated.

3) The quantum efficiency can be written as follows:

$$EQE=\frac{n_{stored}}{n_{generated}}$$

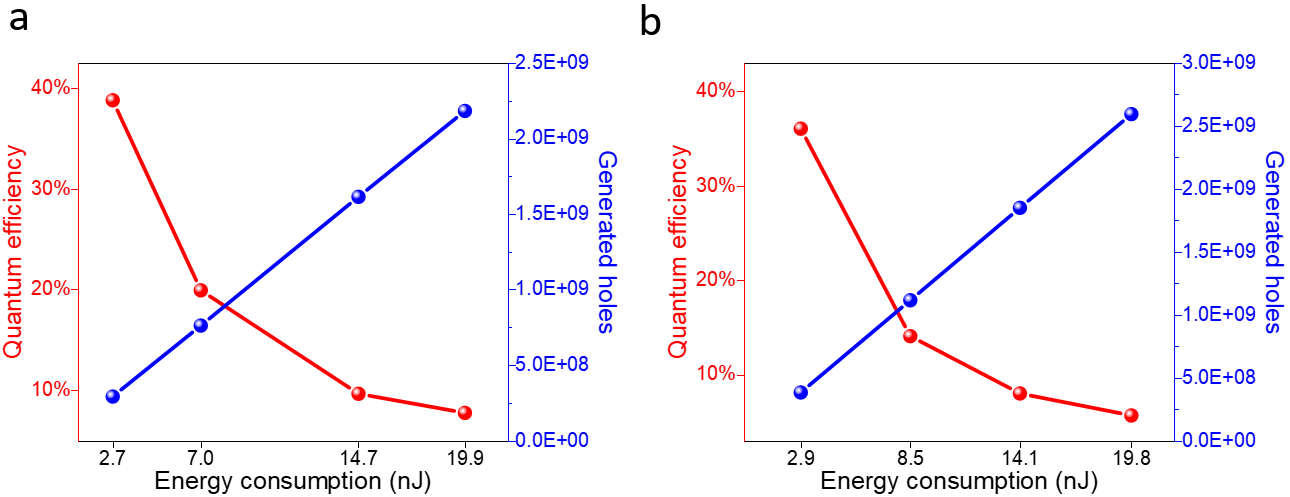


**Fig. S28 | The quantum efficiency and generated holes calculation of critical BTBT memory under different wavelengths and incident power.** **a,** 637 nm. **b,** 830 nm.

According to the above discussion, although the infrared photon exhibits low energy, the infinitesimal barrier facilitates the tunnelling process and exhibits high quantum efficiency. However, bandgap limitation and the thin thickness of the InSe layer refine the absorption, especially in the near-infrared range. Thus, although the bandgap of InSe allows for near-infrared detection, the relatively low absorption is becoming a decisive factor that degrades energy efficiency. Coupling with other optical structures, such as meta-surface, Fabry-Pérot cavities, etc., the absorption of 2D materials can be improved^15,16^.

**Section 17.** **Schematic diagram of the reflection imaging system**


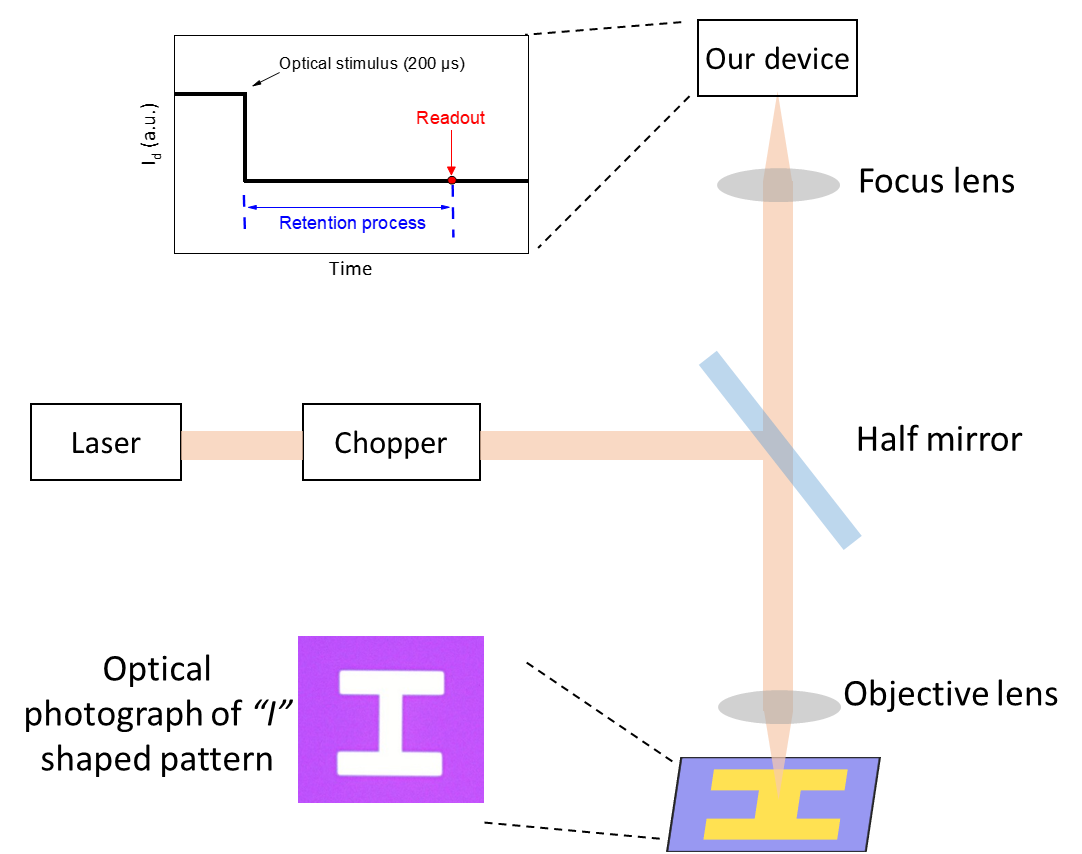


**Fig. S27 | Schematic of the reflection imaging system for moving target tracking.** A 633 nm laser with a 200 μs pulse width is utilized to demonstrate the potential of critical BTBT memory to image objects in an extremely short exposure time, which is the basis for tracking the moving target. Moreover, the current is read out after 0.5 s of optical stimulus to satisfy the inter-frame algorithm. All the “I” shaped imaging results are obtained through experiments.

**Section 18. Evaluation of tracking ability**

For moving target tracking, matching the same objects in different frames is critical. The first step is to find the feature points in the images. Then, we can establish connections between the same object across frames based on these feature points. In this work, the Harris corner detector, a robust algorithm, is used to discriminate the feature points in time-series images. In **Fig. S29,** we extracted the feature points based on critical BTBT memory and other optoelectronic memory results in **Figure 4** through the Harris corner detector. Although there is little difference in the number of feature points, the number of feature points matched through the optical flow method varies greatly. As shown in **Fig. 4f**, only one feature point is matched based on the optoelectronic memory database. In contrast, there are 17 feature points matched based on the critical BTBT memory results, demonstrating that the critical BTBT memory-based neural network has better tracking ability.


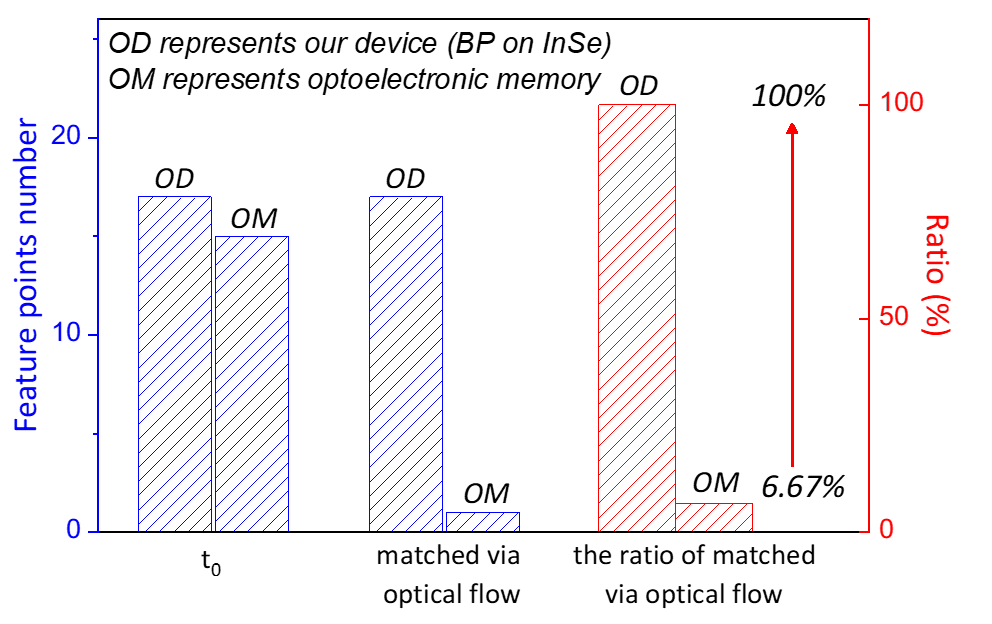


**Fig. S28 | Visualization of feature points based on Harris corner detector.** The blue column represents the feature point numbers at *t_0_*, *t_0_+△t* and the feature points matched across frames, respectively. The red column represents the inter-frame feature points matched ratio; compared to 100% of critical BTBT memory, optoelectronic memory is only 6.67%.

**Section 19. Critical BTBT memory array demonstration**

**Ⅰ. Preparation of 4×4 critical BTBT memory array**

The detailed fabrication process is as follows: large BP and InSe layers are obtained by mechanical exfoliation. A dry transfer is used to ensure a clean interface without introducing impurities. Then, the critical BTBT memory area is defined by EBL and etched by inductively coupled plasma etch (ICP) sequentially. In the process of ICP, Cl_2_ is used as a reactant to etch the heterojunction into 4×4 separate critical BTBT memory in one step; the response time is 30 seconds. After that, the electrodes are defined by EBL again and deposited by thermal evaporation. The electrode consists of 15 nm Cr and 35 nm Au.

Due to the inevitable surface undulations and uneven stresses during the transfer process, the interface contact is poor. Finally, the nitrogen annealing process is performed in the last step to guarantee the robustness of critical BTBT memory arrays and reduce the charge storage caused by non-compactness.


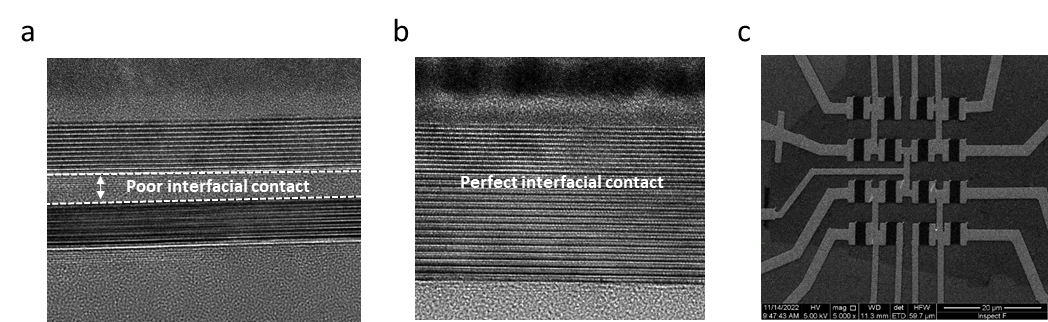


**Fig. S29 | TEM and SEM results of 4×4 IFM array. a,** Cross-sectional TEM image of the overlapped region. No contact between double materials. **b,** Cross-sectional TEM image of the overlapped region, which has been improved by nitrogen annealing, demonstrating a perfect interfacial contact. **c,** The SEM demonstration of critical BTBT memory array, the individual device size is 2 μm×5 μm.

**Ⅱ. Photomemory characterization of fabricated critical BTBT memory array**


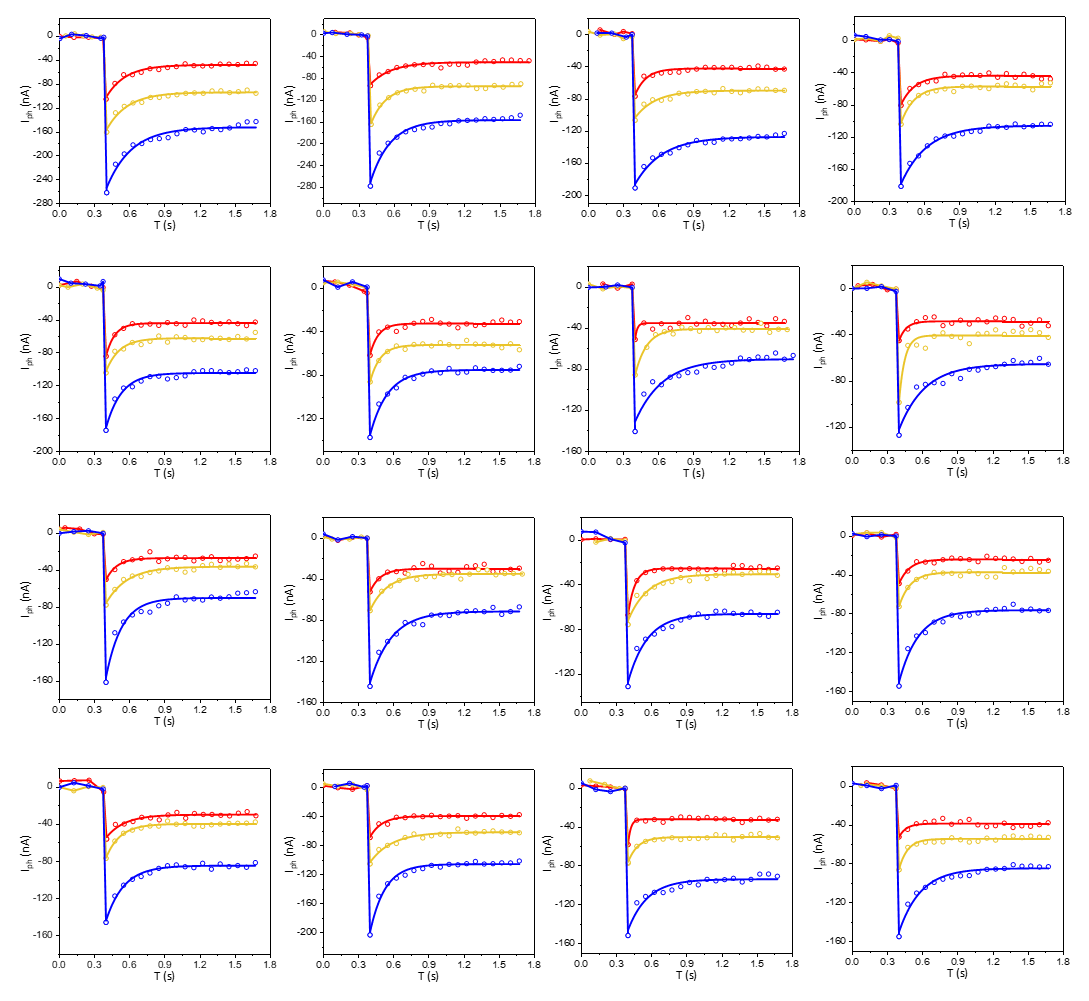


**Fig. S30 | Photoresponse of critical BTBT memory 4×4 array.** Negative photomemory characteristics of the 16 individual pixels in the 4×4 critical BTBT memory array. The laser stimulus duration is 50 μs (red lines), 100 μs (yellow lines) and 500 μs (blue lines). Each device works at *V_d_* = 1 V, *V_g_* = -5 V.

**Section 20. Benchmark of the optoelectronic memories**

| **Materials** | **Wavelength** | **Optical pulse width** | **Mechanism** | **Refs** |
| --- | --- | --- | --- | --- |
| Gr/MoS_2_ | 635 nm | 0.1 s | Photogating | 17 |
| hBN/WSe_2_/Al2O_3_/BP | 637 nm | 100 μs | Floating gate | 18 |
| O_D_-IGZO/O_R_-IGZO | 800 nm | 1 s | Photogating | 19 |
| WSe_2_/hBN/Al_2_O_3_ | 405 nm | 10 ms | Photogating | 20 |
| VO_2_ | 375 nm | 1 s | Phase change | 21 |
| Al_2_O_3_/HfO_2_/Al_2_O_3_/BP | 780 nm | 1 ms | Floating gate | 12 |
| Ir(bt)_2_(acac)/PS-b-P4VP | 525 nm | 700 ms | Photogating | 22 |
| MoS_2_/hBN/Gr | 532 nm | 1 ms | Floating gate | 23 |
| MoS_2_/ hBN /2D-RPP | 532 nm | 1.2 s | Floating gate | 24 |
| WSe_2_/Al_2_O_3_/WSe_2_ | 655 nm | 950 ms | Floating gate | 25 |
| FAPbBr_3_NC/SWCNT | 405 nm | 30 μs | Photogating | 26 |
| PQD/Graphene | 440 nm | 5 s | Photogating | 27 |
| MoS_2_/hBN/Gra | 458 nm | 0.1 s | Floating gate | 28 |
| Gra/SWNT | 405 nm | 100 ms | Photogating | 29 |
| WSe_2_/hBN | 405 nm | 0.5 s | Photogating | 30 |
| BP/InSe | 940 nm (NIR) | 500 ns | BTBT | This work |
|  |  |  |  |  |

**References**

1 Wu, F. *et al.* AsP/InSe Van der Waals Tunnelling Heterojunctions with Ultrahigh Reverse Rectification Ratio and High Photosensitivity. *Advanced Functional Materials* **29**, doi:10.1002/adfm.201900314 (2019).

2 Chen, Y. *et al.* Unipolar barrier photodetectors based on van der Waals heterostructures. *Nature Electronics* **4**, 357-363, doi:10.1038/s41928-021-00586-w (2021).

3 Gao, A. *et al.* Observation of ballistic avalanche phenomena in nanoscale vertical InSe/BP heterostructures. *Nat Nanotechnol* **14**, 217-222, doi:10.1038/s41565-018-0348-z (2019).

4 Miao, J. *et al.* Heterojunction tunnel triodes based on two-dimensional metal selenide and three-dimensional silicon. *Nature Electronics* **5**, 744-751, doi:10.1038/s41928-022-00849-0 (2022).

5 Zhao, S. *et al.* Highly Polarized and Fast Photoresponse of Black Phosphorus-InSe Vertical p-n Heterojunctions. *Advanced Functional Materials* **28**, doi:10.1002/adfm.201802011 (2018).

6 Lv, Q. *et al.* Interlayer Band‐to‐Band Tunnelling and Negative Differential Resistance in van der Waals BP/InSe Field‐Effect Transistors. *Advanced Functional Materials* **30**, doi:10.1002/adfm.201910713 (2020).

7 Wang, H. *et al.* Repression of Interlayer Recombination by Graphene Generates a Sensitive Nanostructured 2D vdW Heterostructure Based Photodetector. *Adv Sci (Weinh)* **8**, e2100503, doi:10.1002/advs.202100503 (2021).

8 Lee, J. *et al.* Monolayer optical memory cells based on artificial trap-mediated charge storage and release. *Nat Commun* **8**, 14734, doi:10.1038/ncomms14734 (2017).

9 Zubair, M. *et al.* Gate‐Tunable van der Waals Photodiodes with an Ultrahigh Peak‐to‐Valley Current Ratio. *Small*, doi:10.1002/smll.202300010 (2023).

10 Wang, P. F. *et al.* A semi-floating gate transistor for low-voltage ultrafast memory and sensing operation. *Science* **341**, 640-643, doi:10.1126/science.1240961 (2013).

11 Fang, H. & Hu, W. Photogating in Low Dimensional Photodetectors. *Advanced Science* **4**, doi:10.1002/advs.201700323 (2017).

12 Lee, S., Peng, R., Wu, C. & Li, M. Programmable black phosphorus image sensor for broadband optoelectronic edge computing. *Nature Communications* **13**, doi:10.1038/s41467-022-29171-1 (2022).

13 Wang, C.-Y. *et al.* Gate-tunable van der Waals heterostructure for reconfigurable neural network vision sensor. *Science Advances* **6**, eaba6173, doi:doi:10.1126/sciadv.aba6173 (2020).

14 Zhao, Y., Nie, K. & He, L. Modulation of optical absorption properties of monolayer InSe by introducing intermediate impurity levels in the band gap. *Chemical Physics Letters* **824**, 140549, doi:<https://doi.org/10.1016/j.cplett.2023.140549> (2023).

15 Li, Q., Lu, J., Gupta, P. & Qiu, M. Engineering Optical Absorption in Graphene and Other 2D Materials: Advances and Applications. *Advanced Optical Materials* **7**, doi:10.1002/adom.201900595 (2019).

16 Luo, X. *et al.* A review of perfect absorbers based on the two dimensional materials in the visible and near-infrared regimes. *Journal of Physics D: Applied Physics* **55**, doi:10.1088/1361-6463/ac3034 (2021).

17 Roy, K. *et al.* Graphene-MoS2 hybrid structures for multifunctional photoresponsive memory devices. *Nat Nanotechnol* **8**, 826-830, doi:10.1038/nnano.2013.206 (2013).

18 Zhang, Z. *et al.* All-in-one two-dimensional retinomorphic hardware device for motion detection and recognition. *Nature Nanotechnology*, doi:10.1038/s41565-021-01003-1 (2021).

19 Hu, L. *et al.* All‐Optically Controlled Memristor for Optoelectronic Neuromorphic Computing. *Advanced Functional Materials* **31**, doi:10.1002/adfm.202005582 (2020).

20 Wang, S. *et al.* Nonvolatile van der Waals Heterostructure Phototransistor for Encrypted Optoelectronic Logic Circuit. *ACS Nano* **16**, 4528-4535, doi:10.1021/acsnano.1c10978 (2022).

21 Li, G. *et al.* Photo-induced non-volatile VO(2) phase transition for neuromorphic ultraviolet sensors. *Nat Commun* **13**, 1729, doi:10.1038/s41467-022-29456-5 (2022).

22 Islam, M. M. *et al.* Multiwavelength Optoelectronic Synapse with 2D Materials for Mixed-Color Pattern Recognition. *ACS Nano* **16**, 10188-10198, doi:10.1021/acsnano.2c01035 (2022).

23 Wiyanto, L. D., You, B. J., Chiang, L. J., Yang, D. L. & Chen, J. Y. Novel Application of Phosphorescent Material for Non‐Volatile Flash Photomemory and Artificial Photonic Synapse. *Advanced Functional Materials* **32**, doi:10.1002/adfm.202206040 (2022).

24 Lai, H. *et al.* Photoinduced Multi-Bit Nonvolatile Memory Based on a van der Waals Heterostructure with a 2D-Perovskite Floating Gate. *Adv Mater* **34**, e2110278, doi:10.1002/adma.202110278 (2022).

25 Hou, X. *et al.* A Logic-Memory Transistor with the Integration of Visible Information Sensing-Memory-Processing. *Adv Sci (Weinh)* **7**, 2002072, doi:10.1002/advs.202002072 (2020).

26 Hao, J. *et al.* Low-energy room-temperature optical switching in mixed-dimensionality nanoscale perovskite heterojunctions. *Science Advances* **7**, eabf1959, doi:doi:10.1126/sciadv.abf1959 (2021).

27 Pradhan, B. *et al.* Ultrasensitive and ultrathin phototransistors and photonic synapses using perovskite quantum dots grown from graphene lattice. *Science Advances* **6**, eaay5225, doi:doi:10.1126/sciadv.aay5225 (2020).

28 Tran, M. D. *et al.* Two-Terminal Multibit Optical Memory via van der Waals Heterostructure. *Adv Mater* **31**, e1807075, doi:10.1002/adma.201807075 (2019).

29 Qin, S. *et al.* A light-stimulated synaptic device based on graphene hybrid phototransistor. *2D Materials* **4**, 035022, doi:10.1088/2053-1583/aa805e (2017).

30 Xiang, D. *et al.* Two-dimensional multibit optoelectronic memory with broadband spectrum distinction. *Nat Commun* **9**, 2966, doi:10.1038/s41467-018-05397-w (2018).
